# Life and death in early colonial Campeche: new insights from ancient DNA

**DOI:** 10.15184/aqy.2022.79

**Published:** 2022-07-13

**Authors:** Vera Tiesler, Jakob Sedig, Nathan Nakatsuka, Swapan Mallick, Iosif Lazaridis, Rebecca Bernardos, Nasreen Broomandkhoshacht, Jonas Oppenheimer, Ann Marie Lawson, Kristin Stewardson, Nadin Rohland, Douglas J. Kennett, T. Douglas Price, David Reich

**Affiliations:** 1 Facultad de Ciencias Antropológicas, Universidad Autónoma de Yucatán, km. 1, Carr. Mérida Tizimín, Mérida, Yuc, CP97305, Mexico.; 2 Harvard Medical School, Department of Genetics, Boston, MA 02115, USA; 3 Howard Hughes Medical Institute, Boston, MA 02115, USA; 4 Broad Institute of Harvard and MIT, Cambridge, MA 02142 USA; 5 Department of Anthropology, University of California, Santa Barbara, CA 93106, USA; 6 Department of Anthropology, University of Wisconsin, Madison, WI, 53706, USA

## Introduction

**Figure 1.**
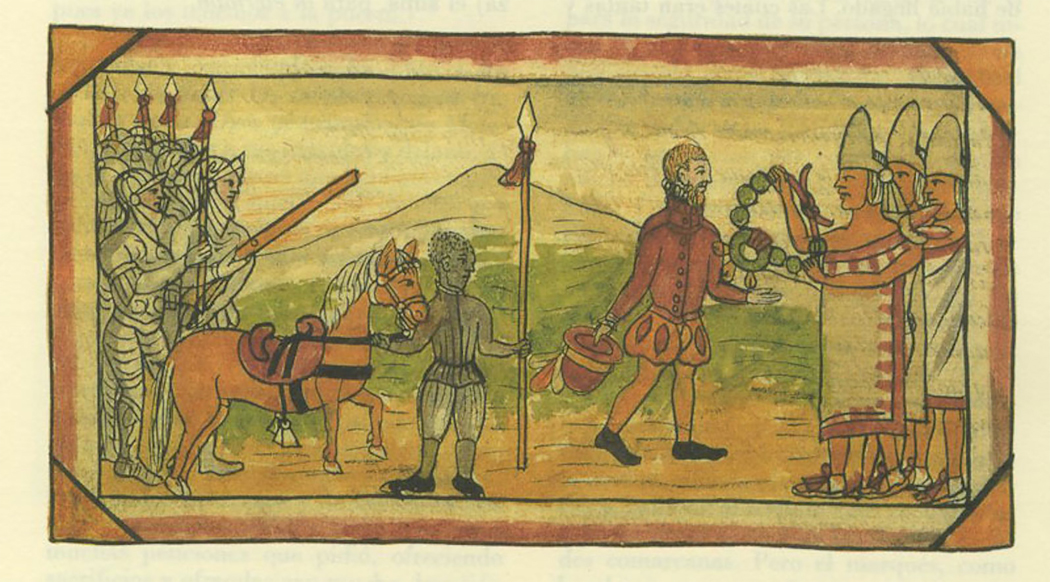
Colonial sources attest that Juan Cortés, slave of the soldier Juan Sedeño, was the first African registered in New Spain. He was part of the military consortium led by Hernán Cortés in 1519 (Bishop Diego Durán; image courtesy of Arqueología Mexicana/Raices).

**Figure 2.**
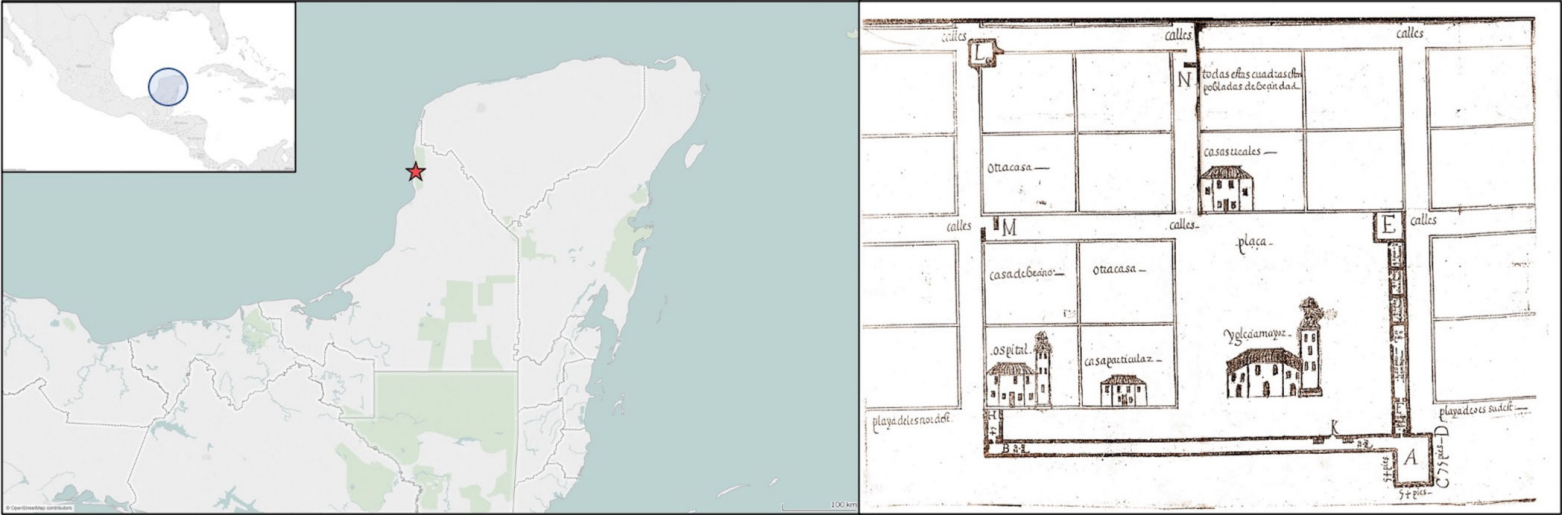
Locations of (left) the town of Campeche (map by the authors) and (right) the old Novohispanic church within the fortified central plaza of San Francisco de Campeche in 1604 (source: Historical Military Service of Madrid, Spain).

After the Spanish conquistadors had conquered Aztec Central Mexico in less than two years after their arrival in 1519, it took them two decades more to secure their rule also over Yucatán and its native Maya population. By the end of 1540 (Figure 1), Conquistador Francisco de Montejo ‘the younger’ finally founded the villa of San Francisco de Campeche as part of the Spanish third colonization campaign in Yucatán (Valencia 1939). This coastal Hispanic town replaced the nearby Maya settlement of Ah Kin Pech, after which it was named (Valencia 1939; Scholes & Roys 1996); Figure 2a). On the central plaza, its first formal church was erected (Figure 2b) to serve “Spaniards, *mestizos*, *mulattos*, blacks, Indian *naboríos* and seven other Indian tribes” (López de Cogolludo 1954). Later in time, this early parish church and its churchyard came to administer all those baptized settlers who weren’t part of any of the surrounding district congregations—visitors, recent immigrants, together with the residents of the houses that lined the blocks surrounding Campeche’s main plaza, an area which from the beginning was reserved for Spanish residents, their servants, and harbor workers (Antochiw 2010; Price *et al*. 2012).

Nestled on the edge of the sea that divides the Americas from Europe and Africa, the early Hispanic town of Campeche, Mexico, thus provides a unique opportunity to examine the intermingling of previously distinct cultures and populations. Declared a UNESCO world heritage site in 2000, its historic center stands as a monumental landmark of the beginnings of Hispanic society. Among the enigmatic findings of Campeche’s early colonial core counts a pristine churchyard that was discovered with the foundations of an early colonial church (Tiesler *et al.* 2010). Some 129 interments were recovered and examined by combining isotopic research with more conventional methods in archaeology and physical anthropology; this article provides new whole genomic ancient DNA (aDNA) data for 10 of those individuals and shall be discussed jointly with the previously published datasets.

### Exploring Campeche’s Early Colonial Population at the Burial Ground from its Main Plaza

**Figure 3.**
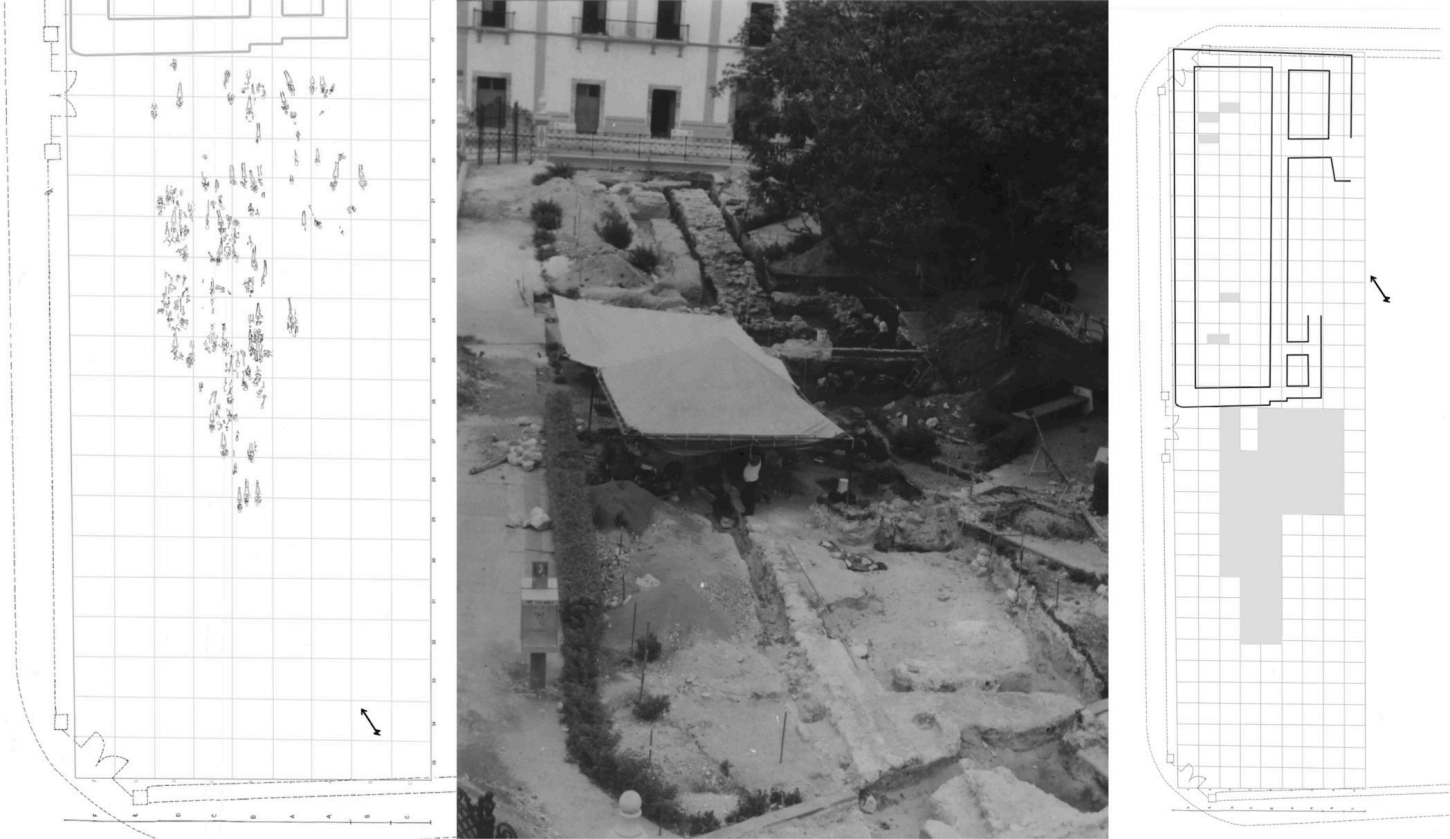
Explorations at the main plaza of Campeche: left) burial distribution; middle) excavation in progress in 2000 (photograph courtesy of Instituto Nacional de Antropología e Historia (INAH)); right) grid of excavation area and test pits (courtesy of INAH & Laboratorio de Bioarqueología/Autonomous, University of Yucatan).

Campeche’s early colonial churchyard was likely established only a few years after the town was founded and was in use until the present cathedral of Campeche replaced it in 1680 (Tiesler *et al.* 2010). The foundations deteriorated together with the associated burial ground that it had once administered following the demolition of the early parish church. It was not until the year 2000 that an archaeological rescue operation led by Mexico’s Instituto Nacional de Antropología e Historia (INAH) brought back the memory of the early parish church and its deceased colonial parishioners, whose bodies once lined the rows of its churchyard. Archaeological mapping, recovery, and cleaning of a total of 129 retrieved burials (Figure 3; see S1), along with subsequent combined bioarchaeological, archaeometric, conventional archaeological, and historical record analyses indicated that this burial ground had been in use for at least 140 years, from the mid 16^th^ century to the late 17^th^ century (Tiesler *et al.* 2010; Price *et al*. 2012; see also S1, S3, and S4). The densely packed burial area contained simple extended interments, following Catholic tradition. Most of the articulated skeletons showed disturbance by subsequent occupations and a number of the burials featured purely co-mingled remains. Only two individuals were found with associated personal items.

Conventional and archaeometric analyses were coordinated at the Laboratory of Bioarchaeology and Histology at the Autonomous University of Yucatán, Mexico (see S2). Macroscopic, histological, and molecular studies, using standard metric and non-metric procedures (S1-S4), were combined to contribute to the reconstruction of age-at-death, sex estimation, geographic provenience, biological ancestry, living conditions, health, nutrition, and artificial body modifications in this cemetery population (Tiesler *et al.* 2010). Macroscopic assessments revealed both male and female adolescents and adults, the majority of whom died before reaching mature adulthood. Their collective examination points to elevated rates of metabolic and inflammatory conditions, as evidenced by cranial porotic hyperostosis, dental enamel hypoplasia, and periosteal reactions. Developmental physiological stress seemed to be a common feature especially among the local population segments, which even surpassed those of their Prehispanic Maya peers (Tiesler *et al.* 2015; Cucina 2010).

**Figure 4.**
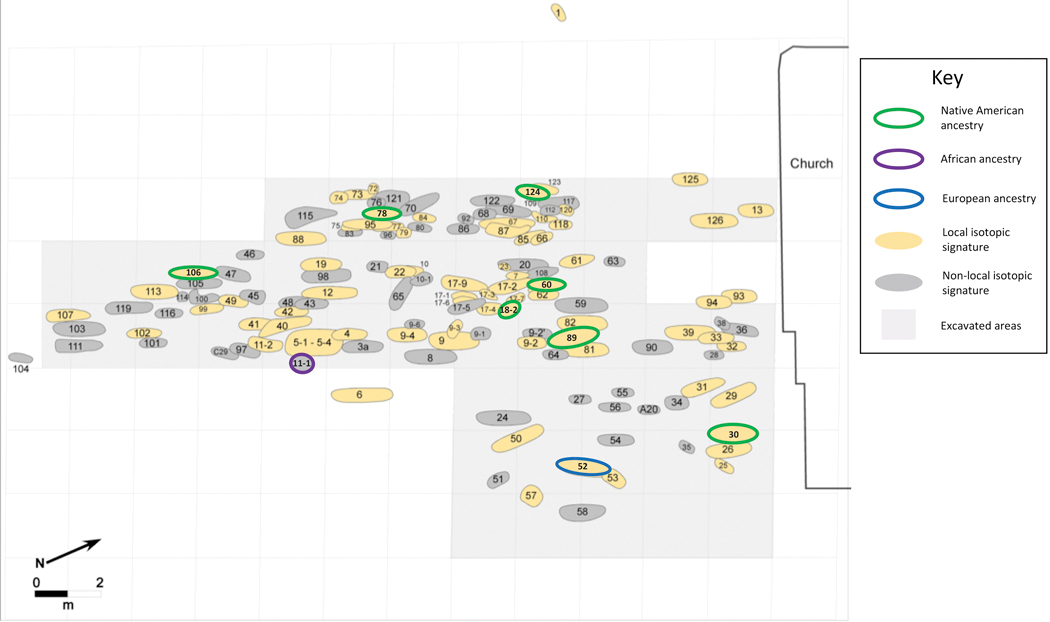
Schematic plan of grave locations surrounding the early colonial church on Campeche’s central plaza (figure by the authors).

The geographic origin and potential biological affinity of the individuals from the Campeche cemetery were also previously examined through analysis of isotopic values (conducted by a team at the University of Wisconsin in Madison, led by T. Douglas Price, see Supplementary Material S3), body modifications, and dental morphological traits scored on the permanent dentition (see S2). The profiling of Sr and O isotopes and comparisons with respective baseline data suggested that a considerable segment of the population consisted of first-generation immigrants from near and far, including West Africa for at least a dozen individuals (Figure 4). Results from isotopic analyses correlate with biomorphological assessments—all individuals with signs of cultural head shaping were determined to be locals, while four cases of dental chipping, an African practice, were determined as non-locals (Price *et al*. 2006). The combination of all lines of evidence culminated in an edited volume (Tiesler *et al.* 2010) and a major article (Price *et al.* 2012). The combined understanding of individual life-course profiles and layered assessments of the overall burial population from the churchyard have fostered an integrated although hypothetic understanding of life and death during the early Colonies at Campeche and indicate that individuals of Native, European, and African origin were buried side by side and potentially together with population of admixture. The following study takes up the state of exploration after a decade by adding novel paleogenomic information.

### Paleogenetic Examination

To further examine the early Hispanic cemetery and population of colonial Campeche, we selected ten individuals for aDNA analysis. The first author (Tiesler) had previously attempted unsuccessfully to generate aDNA data from the Campeche cemetery. However, this was prior to the exponential growth in aDNA studies that began around 2015 and the discovery that the petrosal portion of the temporal bone often generates many-fold more data than other skeletal elements (Pinhasi *et al.* 2015). With these developments, Tiesler decided to once again attempt aDNA analyses and selected the 10 petrous portions that visually had the best preservation in the Campeche series. The goals of our sampling were to: 1) determine if it was possible to generate genome-wide data from the petrous portions of individuals buried in the Campeche cemetery; 2) compare results from aDNA to previously gathered biomorphological and biomolecular data; and 3) explore any patterns evident in the aDNA data. Permission to study the individuals was granted by INAH’s Consejo de Arqueología (S5).

**Figure 5.**
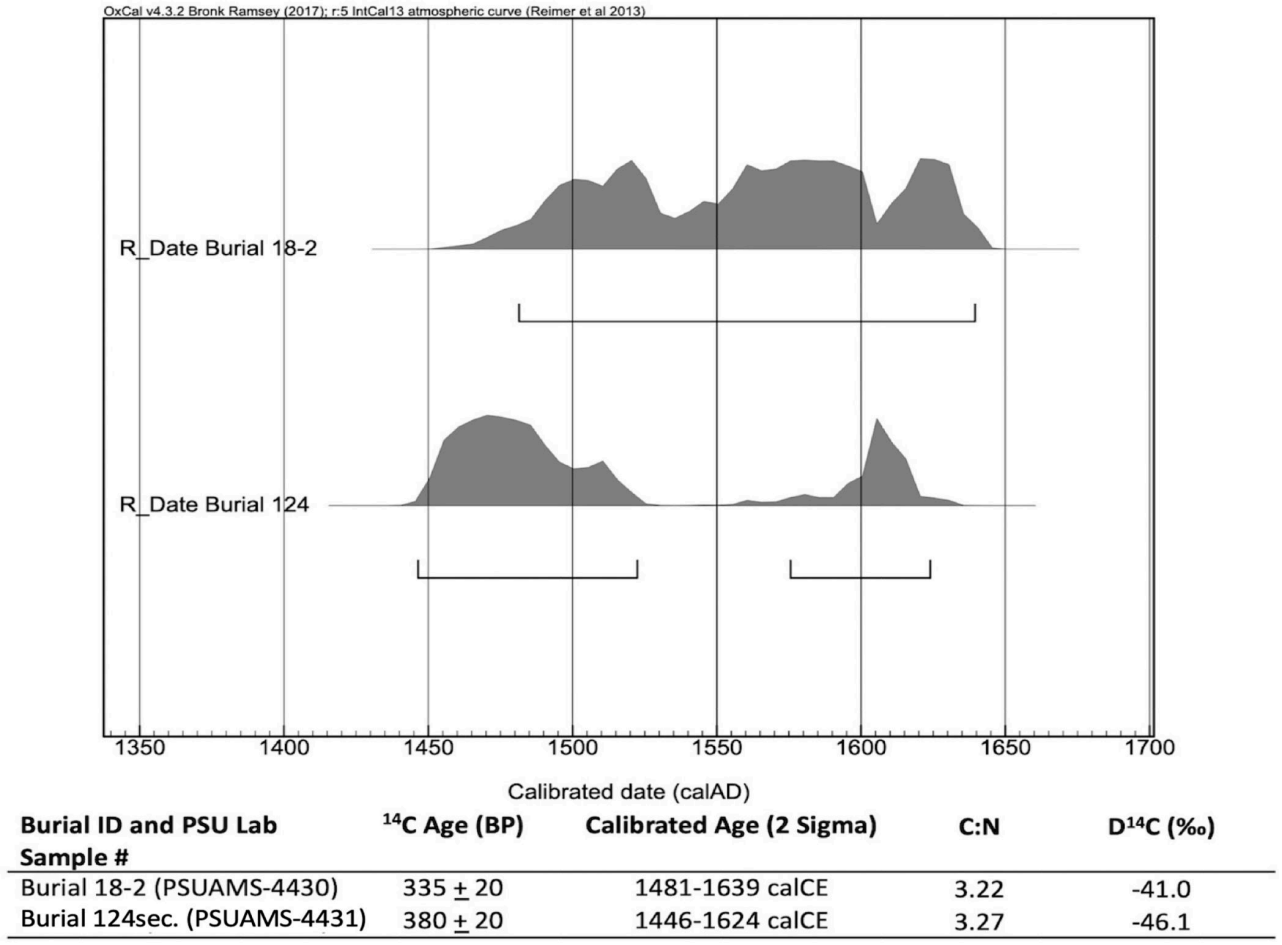
Plotted radiocarbon distributions of two individuals from the Campeche churchyard dated as part of this research (dates calibrated in OxCal v4.3.2, using the IntCal13 atmospheric curve; Reimer et al. 2013; Bronk Ramsey 2017) (figure by the authors).

We met our first goal and generated working data from all 10 of the petrosal portions selected for analysis (we also radiocarbon dated two individuals, see Figure 5). Table 1 provides basic data from analysis of these individuals (additional details are provided in Supplement S4 and Online Dataset 1). We performed a suite of standard aDNA analyses to achieve our second and third goals. Determinations of genetic sex, mitochondrial haplogroups, and Y chromosome haplogroups were made on all individuals. No close genetic relatives were detected amongst the 10 individuals (up to 3^rd^ degree relatives). Mitochondrial lineages for most individuals were either B2 or A2 (haplogroup lineages remain unchanged when restricting to reads with a characteristic damage signal of ancient DNA), which are both common among Indigenous Maya populations (Gonzalez-Martín *et al.* 2015; Söchtig *et al*. 2015). Notable mitochondrial haplogroup outliers are individual Burial 11–1, who has an L3 mitochondrial haplogroup and Burial 52–1, who has an X2 haplogroup. These haplogroups are most common in African and European populations respectively, which correlates with whole genome data for these individuals.

**Figure 6.**
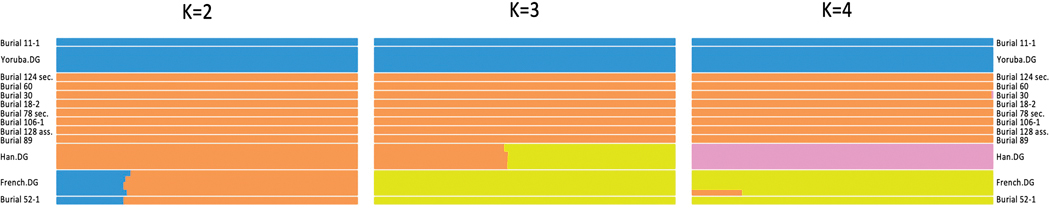
Unsupervised ADMIXTURE clustering plots of the 10 individuals relative to highly diverged modern populations (for modern sample information, see Table S3 in the OSM). The small proportion of Han-related ancestry detected for Burial 30 at K = 4 is statistical noise due to the much lower dataset size (only approximately 40 000 SNPs) for this individual. The Native American-related ancestry detected for the modern French DG individual at K = 4 is also probably an artefact of the data (figure by the authors).

**Figure 7.**
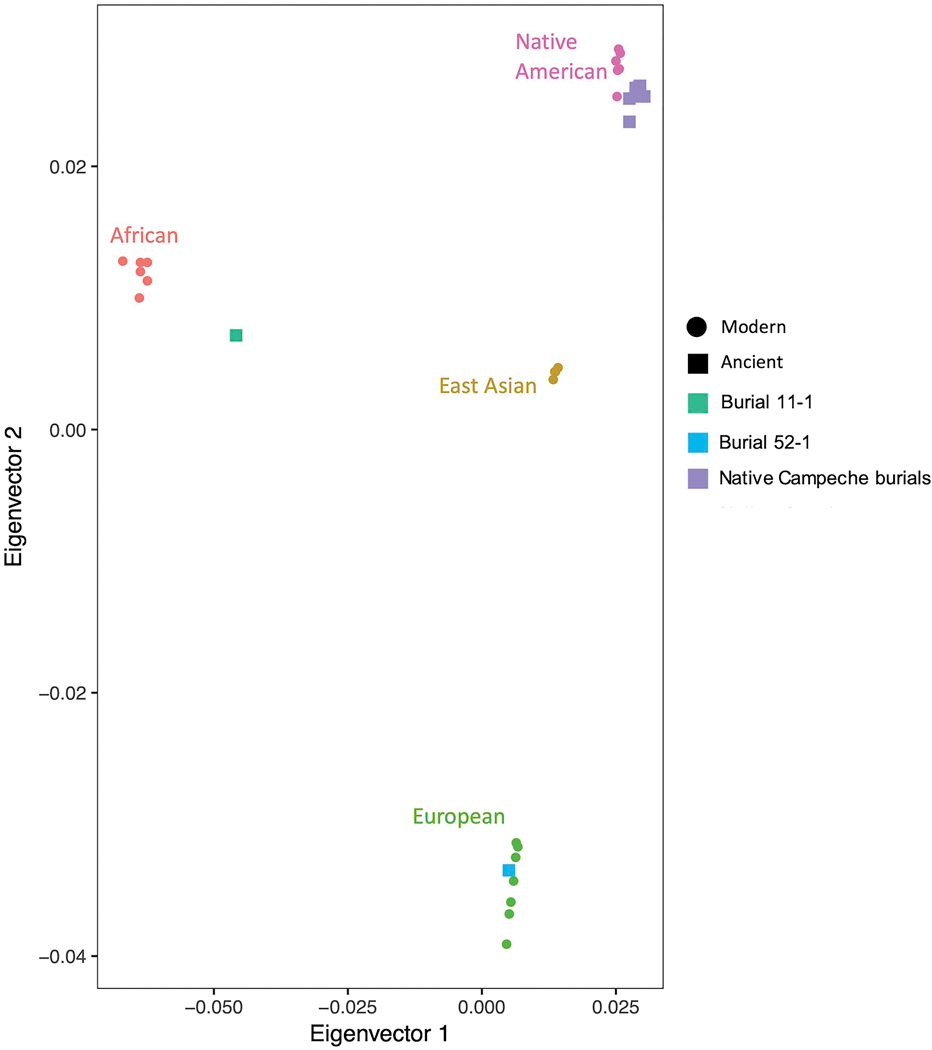
Principal components analysis of ancient individuals projected onto the principal components inferred from modern individuals consisting of Han Chinese, African (Yoruba and Mende), European (French and Finnish) and Native American (Pima, Chane and Mixe) individuals (for modern sample information, see Table S3 in the OSM).

We performed ADMIXTURE clustering analysis (Figure 6), principal component analysis (PCA) (Figure 7), and qpAdm analysis (Online Dataset S2) to provide insight into the population structure of the Campeche individuals and their relationship with previously reported data. These analyses reveal that while the majority of the individuals cluster together as an unadmixed Native Campeche population, there are outliers, including one un-admixed European individual and one un-admixed African individual. We also conducted *qpAdm* analysis to model formally the ancestry of the Campeche individuals. We attempted to model each of the individuals as a mixture of Mixe, Yoruba, or French-related ancestry. These well-studied modern groups have a long history of genetic isolation from each other with little mixture from other populations (prior to European colonization) and therefore allowed us to test if an ancient individual’s ancestry is more correlated with African, European, or Native American populations. We found that the individuals had ancestry proportions consistent with being entirely European (Burial 52–1 individual), entirely sub-Saharan African (Burial 11–1 individual), or entirely Native American (the remaining 8 individuals) (Online Dataset S2).

**Figure 8.**
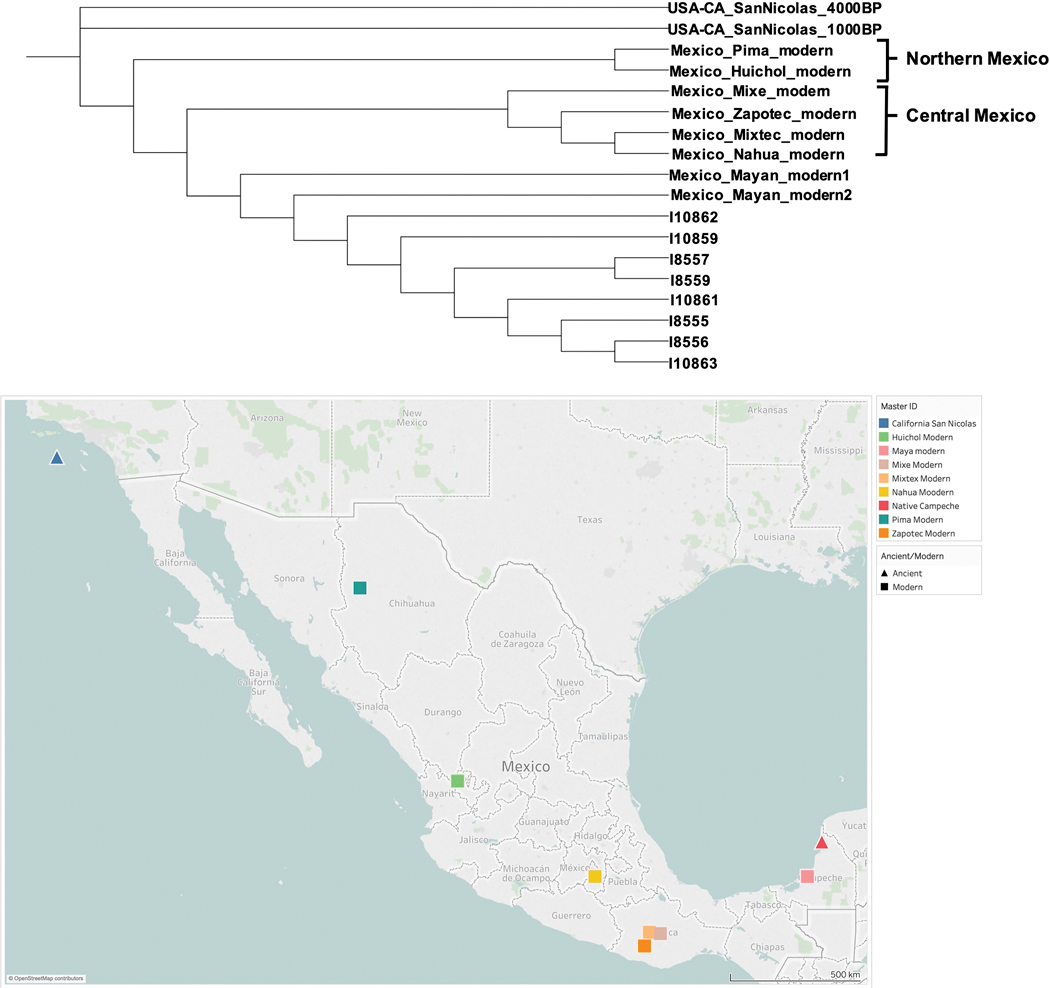
Neighbour joining tree from outgroup-f 3 statistics (above), and location of modern and ancient individuals used (below). Ancient Campeche individuals are most closely related to modern Maya, the group in closest geographic proximity (figure by the authors).

In a further step, we created a neighbor-joining tree with “outgroup”-*f*_*3*_ statistics (Raghavan *et al.* 2014) of the form *f*_*3*_*(Mbuti; Pop1, Pop2*), and found that the Campeche individuals were all more closely related to modern Maya individuals from southern Mexico than to Indigenous groups from central or northern Mexico (Figure 8). This suggests that they were from nearby the region rather than further away in Mesoamerica.

To determine if there is any detectable substructure in the eight individuals consistent with Native American ancestry, we computed statistics of the form [D(*Campeche.sample.A*, *Campeche.sample.B; Campeche.sample.C*; *YRI.SG*)]. These statistics examine whether a test individual “Campeche.sample.C” shares more genetic alleles with “Campeche.sample.A” or “Campeche.sample.B”, using West Africans from Nigeria as an outgroup baseline (YRI.SG). Of all 252 (=9*8*7/2) unique statistics of this type involving comparisons of the individuals inferred to have broadly Native American ancestry, the largest |Z|-score is 2.63, which is not significant after correcting for the number of hypotheses tested (p=0.0087, Bonferroni corrected to p=0.89). Thus, the eight individuals with typical Native American ancestry are unstructured to the limits of our resolution.

### Individual profiles

The majority of the ten individuals under study—all but Burial 11–1 and Burial 52–1— have local Indigenous population genetic profiles. ADMIXTURE analysis at K=4 distinguished the Native Campechean individuals from highly diverged modern populations, (Figure 5), they plot on the Native American portion of the PCA (Figure 6), and they can be modeled as unadmixed Native Americans in *qpAdm* analysis (Online Dataset 2). Additionally, these eight individuals have either A2 or B2 mitochondrial haplogroups, which are the two most common haplogroups in modern Maya populations (Gonzalez -Martín *et al.* 2015; Söchtig *et al*. 2015). The Y chromosome haplogroups (Table 1) for males are also typical of Native American populations.

All eight individuals with Indigenous ancestry had local isotopic signatures ( Price *et al.* 2012). These individuals were excavated from different areas of the burial ground, most showing disarticulation or other sorts of disturbances from post-funerary intrusions in an increasingly packed cemetery (Figure 2; see Figure S1 and S2). Extended on their back, each body had been interred in a simple, narrow pit with the head placed towards the setting sun. This was the churchyard’s uniform pattern of Catholic primary deposition, reflecting Hispanic tradition of the early colonies (Tiesler & Zabala 2010). The burial arrangements and location of all eight Native individuals suggests that they had been baptized in order to be admitted to the sanctified grounds after death. A number of the remains showed signs of chronic periostosis in the inferior extremities, porotic hyperostosis, and enamel hypoplasia in the skull. Additionally, given the diagnostic macroscopic and histological features of postcranial segments (see S1), Burial 78 (either the individual studied for this paper or another individual contained within this multiple commingled burial) suffered from venereal syphilis (Rodríguez Pérez 2010). The sclerotic (dense) bone quality of its left fibular bone and the evocative histomorpological traits, namely delimitation banding (*Grenzstreifen*) and pillow formation (*Polster*), suggest a long sequence of repetitive active episodes of venereal treponematosis (Schultz 2003).

Two additional individuals we examined have entirely non-Native ancestry. Previous assessment of Burial 11–1 suggested this individual was a female of African ancestry who died at a young age, probably in her early twenties (similar to most first-generation African immigrants in the overall Campeche series) (Tiesler *et al.* 2010). Her strontium isotopic ratio was one of the highest measured in the graveyard, indicating that she was not born in the Yucatán peninsula. She also had stable C and N isotope values that suggest C3 plants (like rice) constituted most of her childhood diet, consistent with an earlier residence somewhere in Western Africa (Price *et al.* 2012). Her skeleton was found disarticulated and incomplete, indicating that she had been reburied. Her final resting place was 17m southwest of the main church entrance (Figure S2). This area was among the outer segments of the burial ground, the burials of which were closer to the ground surface than those in areas that were closer to the church foundations. The more outlying areas are likely to have harbored the latest interments of this graveyards, dating to the last decades of the cemetery’s use in the seventeenth century (Tiesler *et al.* 2010).

Genome-wide and mitochondrial (L3 haplogroup) data for Burial 11–1 are consistent with African origin (Figures 5–6 and Online Dataset 2). The high resolution genetic analysis allows us to confidently determine that Burial 11–1 had negligible European or Native American ancestry (S5). While additional statistical analysis did not reveal a specific geographic origin for Burial 11–1 (S5), we could determine her ancestry was broadly West African.

**Figure 10.**
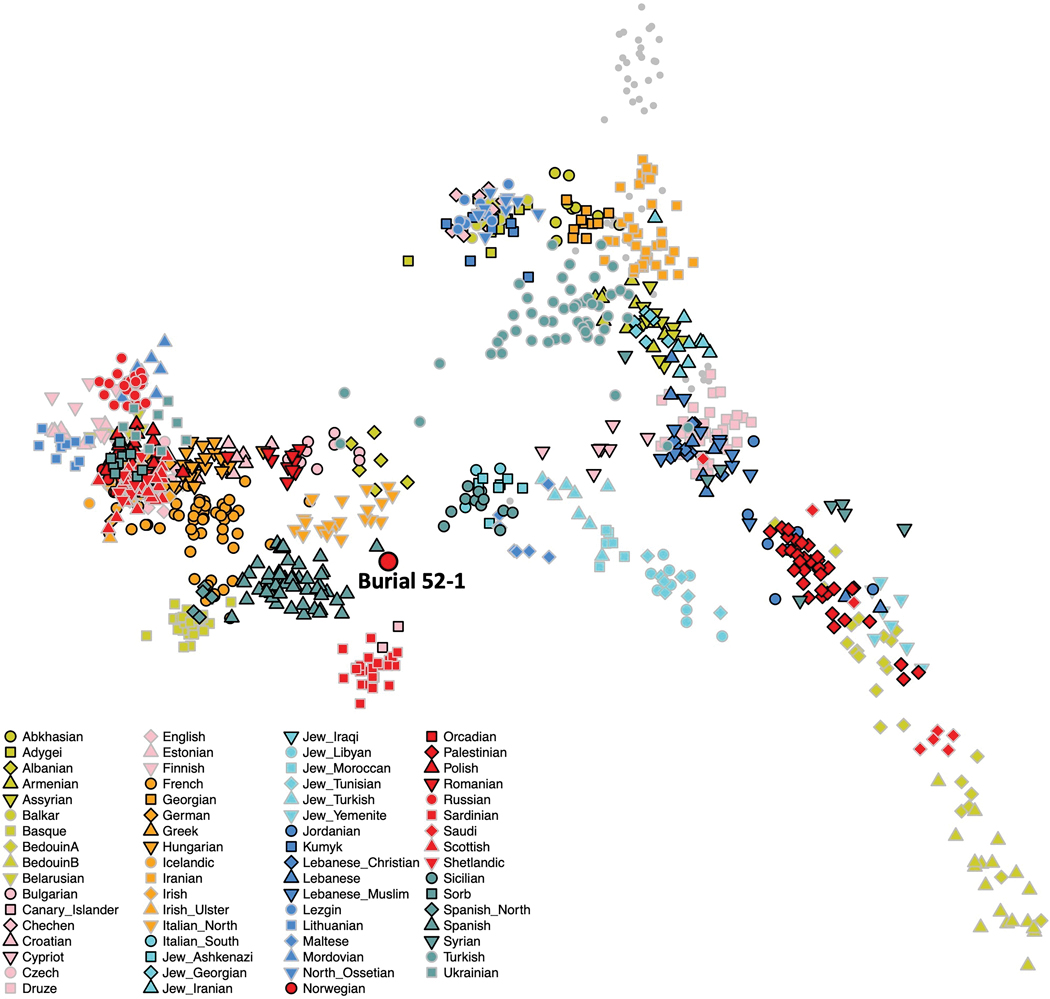
Burial 52–1 projected onto the Eurasian PCA from Lazaridis et al. (2016: 420). This demonstrates that Burial 52–1 is genetically closest to individuals from modern-day Spain and northern Italy, suggesting an overall north-western Mediterranean ancestry (figure by the authors, based on data from Lazaridis et al. (2016)).

Previous assessments identified Burial 52–1 as an adult middle-aged male buried adjacent to the church wall (Tiesler *et al.* 2010). He was likely one of the first generation to be buried with the growing ranks of deceased in the expanding cemetery of the rapidly growing town. Concave wear between his right canine and first premolar indicates that a round abrasive object was habitually kept at the same spot between the two arches, suggesting regular use of a tobacco pipe (Figures 9a,b). His dental epigenetic traits were suggestive of sub-Saharan African or European ancestry (Scott and Turner 1997), but his ^87^Sr/^86^Sr isotopic signature was not. The ^87^Sr/^86^Sr value from bone apatite of this individual was consistent with the local Campeche values, suggesting he had lived at or near Campeche for some time before his death. However, lead isotope values from the enamel of this individual’s tooth suggest he was born and spent at least part of his childhood in Europe, likely southwestern Spain, which correlates with ancient DNA results (Price *et al.* 2012). Genetic analysis indicated this individual had unmixed European ancestry; projection of this individual onto the PCA produced in Lazaridis *et al.* (2016) suggests this individual has western Mediterranean ancestry (Figure 10). He had a Y haplogroup of I2a2a1b, common in European populations. Thus, this individual was most likely a European colonist who lived and died in Campeche, but spent at least part of his youth in Europe, particularly Spain.

#### Burial 128 ass.

We discuss Burial 128 ass. here not because of genetic ancestry—our analyses suggest this individual is unambiguously Native—but because of the inconsistency of our new genetic analyses with previous macroscopic and isotopic analyses of this burial. This was a disturbed but mainly articulated skeleton, which was accompanied by isolated segments of at least three more individuals. It is of note that segments of the cranium were found associated with the mandible but were not articulated and were therefore not traced in the taphonomic skeletal drawing (Figure S3). Conventional analyses determined the articulated individual — which we identify in the following as Burial 128–1— as probably male on the basis of robusticity, pelvic proportions, and the elevated overall body length that was measured and calculated on-site (Tiesler & Zabala 2010). Previous isotopic study (Price et al. 2012) also demonstrated that like Burial 52–1, Burial 128–1 had a (femoral) bone apatite 87Sr/86Sr value that is consistent with local Campeche values and therefore Campeche residence during his last years of life. However, the carbon isotope ratios in tooth enamel, formed earlier in life, point to a dietary regimen that lacked C4 plants and was very different from the typical Campeche diet. Additionally, the lead isotope values of this adult (measured in one enamel sample from the lower, articulated dental arch), are consistent with residence in southwest Spain during early childhood. Like Burial 52–1, these values, along with interment inside the church (likely denoting special status), led Price and colleagues (2012) to classify the main individual of Burial 128 as a European migrant to the America (see Figure S3b).

Unlike Burial 52–1, however, paleogenetic results are discrepant with both the isotopic signatures and the macroscopic sex estimation in the case of Burial 128; our present analyses of the petrous portion —bagged and labeled in the field as part of this individual— to have unadmixed Native ancestry. We currently have no conclusive interpretation reconciling these contradictions other than that the sampled petrous portion, although recorded in the field as contiguous to the mandible and the postcranium, may belong to a different, isolated adult remain (labeled in this work correspondingly as ‘Burial 128 ass.’). This highlights a methodological pitfall when using spatial association and morphological likeness to individualize disturbed assemblages like this one. It also demonstrates how genetic analyses can make important contributions to the archaeological interpretations of highly commingled assemblages, such as the one studied in this work.

## Discussion

There are noteworthy trends amongst the ten Campeche individuals from whom we obtained ancient DNA. We identified three distinct genetic groupings. Most were Indigenous “Campechanos”; however, we also documented one African female and a European male. None of the individuals within these groups have evidence of admixture from one another, despite living in close proximity and being buried in the same mortuary ground. While our sample size was relatively small, this does come somewhat as a surprise, because the bodies had been recovered from diverse burial sectors with a continued mortuary occupation over 140 years, spanning some five to seven generations. Additionally, though previous ancestry determinations were made based skeletal and archaeological assessments, our selection of individuals to study was made based on skeletal preservation. And, as demonstrated in Table 1, most of the previous assessments were uncertain or indeterminate. In other words, our sampling strategy was not designed so that we intentionally studied individuals we thought were likely to harbor only one particular ancestry component based on phenotype or archaeological context. Another reason lack of evidence of admixture was unexpected was that it is unlikely that most of the individuals died during the years of initial settlement because almost all were recovered from areas in the cemetery more distant from the church (except for Burial 30 in the first row off the church facade and Burial 128 ass.).

The generation of new aDNA data highlights the value of comparing results among different datasets. Co-analyzing distinct datasets can capture the wide-ranging dynamics of a newly globalized world and generate perspectives about the past that are absent in historical accounts (Tiesler *et al.* 2010). This is especially true for colonial society, in which the voices of Natives and Africans, and of children and women, tend to be harshly underrepresented, leading to a skewed perspective on the past. While multiple studies have combined archaeology, isotopes, and DNA data to examine prehistoric Eurasia, few such studies have been successfully conducted in the Americas and especially so the Maya Lowlands, where poor tissue preservation has hampered aDNA acquisition. Thus, our ability to generate robust paleogenomic data is very encouraging, as combining these data with other lines of evidence will continue to be particularly informative at Colonial-era sites.

The tension with historical accounts is apparent in our study of the early colonial cemetery, where settlers from different parts of the world and social sectors shared its grounds in close proximity. Spanish historical records only inform categorically that, when founded in 1540, the villa of Campeche harbored 30 “residents”, with some 40 residents by 1562, and 100 in 1615. These “residents” are implied to have been Spaniards, either newcomers or local residents born in the colonies, whereas natives, free blacks, and *mulattos* were not usually considered as part of the resident population as they lived at the edges of town centers, in so called *barrio* neighborhoods (Antochiw 2010; Tiesler & Zabala 2010).

No systematic information exists on the segregated or un-segregated nature of burial grounds in New Spain, although all baptized parishioners were admitted to the sanctified burial grounds (Tiesler & Zabala 2010). However, in practice, circum-Caribbean Novohispanic urban churchyards were typically segregated according to status and ethnicity (Koch 1983; Stojanowsky 2005). Namely, the “casta” segregation of burial spaces followed the separation among ethnically distinctive neighborhoods. In the Hispanic town grids of New Spain, different “castas” would live and die in their specific parishes, also called “barrios.” Immensely contrasting economic and living conditions characterized the social groups settled according to this “barrio” system (Martínez 2006). This situation prevailed in colonial Campeche itself, once its ethnically segregated neighborhoods with their respective churches and churchyards were in place. Further burial segregation could be expressed architecturally and by internal spatial divides (Jaén *et al.* 2017; Pérez-Castro 2019).

While historic documents paint a picture of social demarcation and segregation, our paleogenetic research demonstrates and confirms previous findings (Tiesler *et al.* 2010) that individuals from populations segregated in most other aspects of life were indeed buried in the same Catholic cemetery, which in this case displays overcrowding with a general lack of organization. Most of the deceased seem to have been interred in austere earthen pits, often reoccupied soon afterwards.

This study also highlights how genetic inferences can sometimes be in conflict with other lines of study, such as dental ethnic assignation, conventional sex determination, isotopic analyses, or macroscopic assignment of skeletal individuals in commingled and otherwise disturbed assemblages (such as the case of Burial 128 in this study). Regarding conventional sex determinations for the cemetery burials, only rough determinations could be made for six of the individuals and could not be made at all for the other four (Table 1) due to commingling and lack of skeletal preservation). However, genetic determinations could be made for all 10 individuals (Table 1). We view such instances as opportunities to re-evaluate discrete lines of evidence from different fields to provide a more critical and therefore robust understanding of the past.

Ancestry assessments founded on the classification of non-metric dental traits appear to be less secure than genetic assessment. Our results show some of the previously published biomorphological ancestry assessments to be inconsistent with paleogenetic ancestry. An additional discrepant observation relates to the nature of poorly preserved, disturbed secondary assemblages, especially when the sampled material came from different anatomical parts (such as dental arcades and petrous portions of Burial 128). Conversely, macroscopically determined cultural body modifications suitable for discerning origins, such as dental chipping or filling and head-shaping, were consistent with the paleogenetic results, although they were of less diagnostic value.

The quality of aDNA preservation produced in the study is also of note. Until recently, acquisition of high numbers of single nucleotide polymorphisms (SNPs) from individuals buried in tropical environments, such as Campeche, was difficult and rare as previous failed attempts demonstrate. Yet we were able to produce working genomic data for all individuals in this study, with SNP counts of more than 500,000 for all but one case; moreover, the recovered genetic material is relatively uncontaminated by modern DNA and show the typical damage profiles for ancient DNA samples (Supplementary Figure S4). All genetic data in this study were procured through processing of disarticulated petrous bone in an ancient DNA clean room (see Methods section). These results highlight the effectiveness of using the petrous bone (Pinhasi *et al.* 2015, 2019; Hansen *et al.* 2017), which has allowed for the acquisition of ancient DNA data from skeletal remains that previously failed due to contamination from modern DNA or poor preservation.

## Conclusion

In this study we combined new paleogenetic data from ten individuals with previously acquired data from other more conventional methods in an effort to establish life profiles for individual skeletons. These analyses now provide key details about the multi-ethnic nature of the people living in early colonial Campeche, where previously disparate groups lived together for the first time under Hispanic rule. Singly or collectively, these genetic profiles add a new “human” layer to our understanding of a crucial era and location of Novohispanic history. The churchyard’s distribution of locals and immigrants of different ancestries confirm prompt and forced integration of all segments into the Novohispanic social fabric. Our analysis of these 10 individuals found no evidence of “mestizaje” (mixing across groups). Our sample set is limited, but this perhaps indicates that though they were buried together, different groups in early colonial urban European hubs maintained some degree of separation in life. Future paleogenetic analyses of the burial populations from other colonial sites such as the San Román or San Francisco parishes shall certainly shed new light on the specific ancestries and of any evidence of biological admixture. While the San Roman neighborhood was established and occupied reportedly by the Nahuas whom Montejo brought from his encomienda in Azcapotzalco, the Parishioners of the San Francisco neighborhood were native Maya (López de Cogolludo 1954). Both Parish churchyards still harbor human remains, which were recovered recently by the INAH. In this same vein, we are confident that future scrutiny of additional individuals from Campeche’s central churchyard will provide an even more nuanced picture of the timing of population mixing in Campeche’s post-contact society. We hope this study provides a starting point for further studies in this direction.

## Supplementary Material

Supplementary Text

Supplementary Table 1

Supplementary Table 2

Supplementary Table 3

## Figures and Tables

**Figure 9a.**
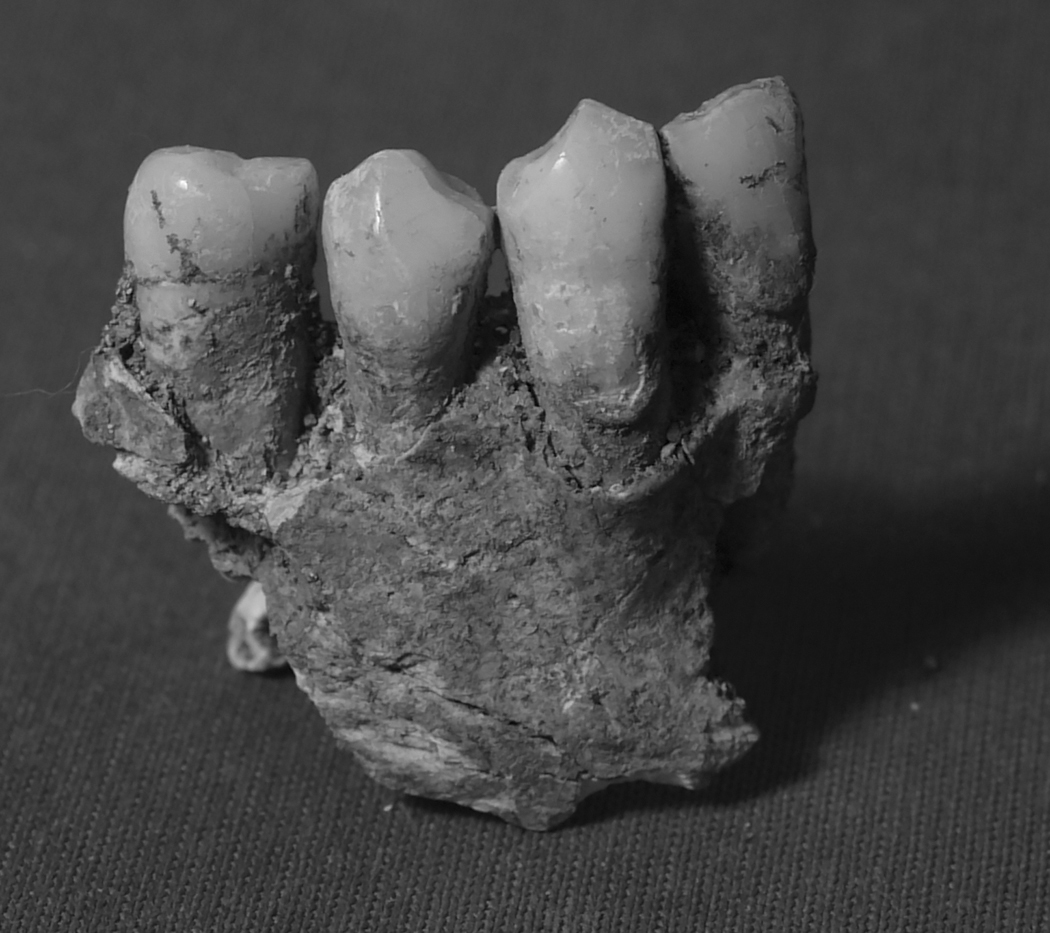
Ethnically distinctive cultural dental modifications: the lower dentition of male Burial 52–1 shows concave wear facets between the canine and first premolar, suggesting regular pipe use.

**Figure 9b.**
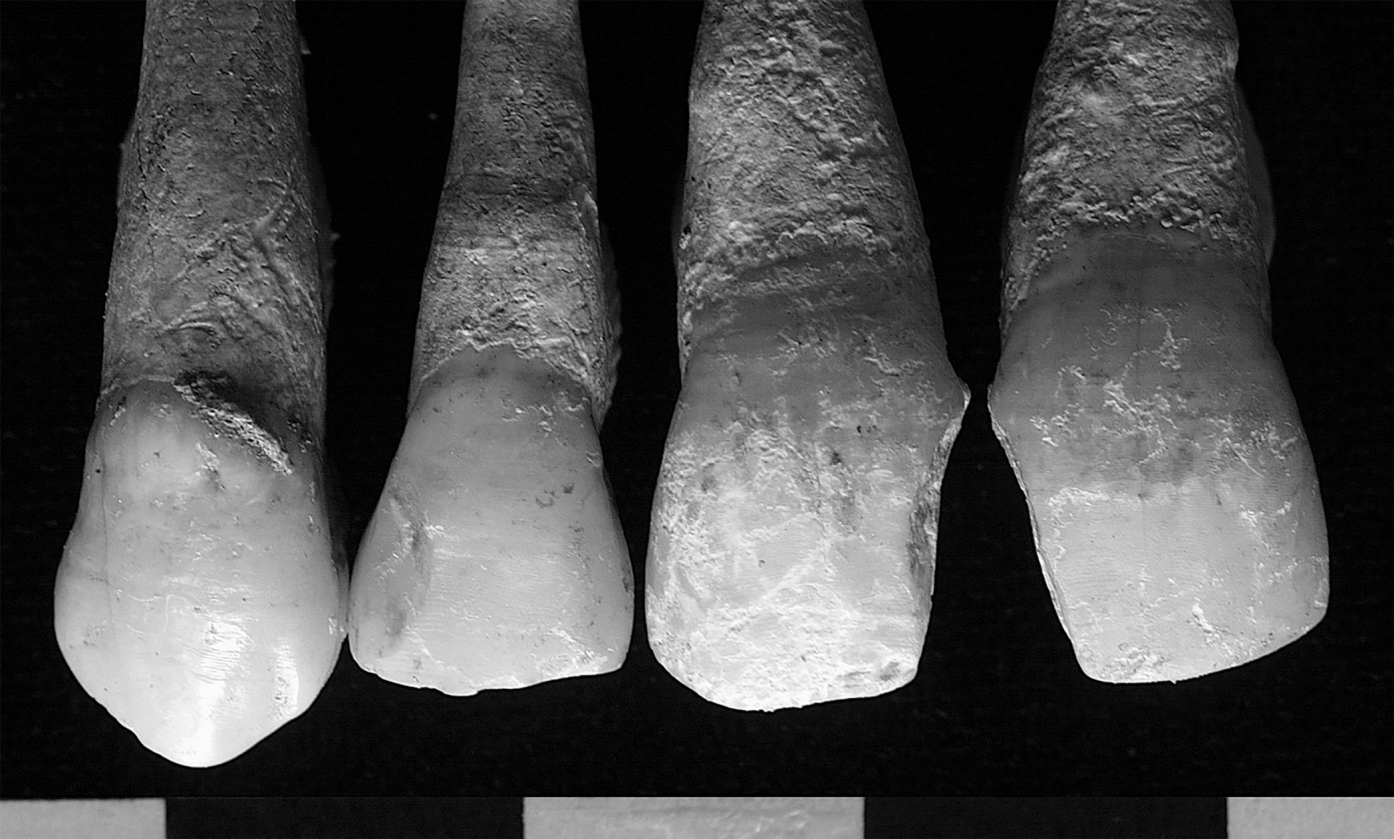
Ethnically distinctive cultural dental modifications: Cleft from chipping in two adjacent central incisors from a collective ossuary within the church perimeter (photographs by the authors).

**Table 1.** Summary data from ancient DNA analysis of 10 ancient Campeche individuals, with previous biomorphological assessments in brackets. See Supplemental Dataset S1 for additional data.

Burial and individual number	#SNPs	Genetic sex compared to [morphological sex]	Genetic ancestry vs. [macroscopic determination of ancestry]	mt Haplogroup	Y haplogroup
Burial 18 ass.	766523	F [M?]	AME [NID]	A2+(64)	..
Burial 30	40807	F [NID]	AME [AME?]	A2r	..
Burial 60	661696	F [NID]	AME [AFR??]	A2+(64)	..
Burial 52.1	845074	M [M?]	EUR [EUR?/AFR?]	X2d2	I2a2a1b
Burial 124 sec.	798004	M [NID]	AME [AME?]	B2	Q1a2a1a1
Burial 78	776783	F [F?]	AME [NID]	A2m	..
Burial 128	730884	F [M?]	AME [NID]	B2	..
Burial 106.1	565042	M [NID]	AME [NID]	A2	Q1a2a1a
Burial 89	669233	M [M?]	AME [NID]	B2+16278	Q1a2a1a1
Burial 11.1	587322	F [F?]	AFR [AFR; AME?]	L3e2b+152	..

Notes: M=male, F=female, NID=not identified, EUR=European, AFR=African, AME=Native American.

## Data Availability

The aligned sequences are available through the European Nucleotide Archive. Genotype data used in analysis are available at https://reich.hms.harvard.edu/datasets. Any other relevant data are available from the corresponding author upon reasonable request.
