# Supplementary material for: Life and death in early colonial Campeche: new insights from ancient DNA
Source: Antiquity. Author manuscript; Available in PMC 2024 Dec 4. (PMC11617036; doi:10.15184/aqy.2022.79)
Supplement: Supplementary Text [file NIHMS2033175-supplement-Supplementary_Text.docx]

Supplementary Information:

**ANCIENT DNA SHEDS NEW LIGHT ON LIFE HISTORIES IN AN EARLY COLONIAL CEMETERY IN CAMPECHE, MEXICO**

**Supplementary materials for this manuscript include the following**:

Supplementary Text S1-S5

Supplementary Figures and Figure Legends S1-S6

Supplement References

Dataset S1—Ancient DNA Results Extended Information

Dataset S2—qpAdm values

Dataset S3—F_3_ Statistics for Burial 11-1

In what follows, we provide detailed information on the archaeological context, physical anthropological analysis of the skeletons, isotopic analysis, radiocarbon dating analysis, and genetic analysis. We note that Supplements S1-S4 present an adapted and abbreviated version of the methods and results presented a decade ago in an edited volume (Tiesler et al. 2010) and an article publication (Price et al. 2012). Supporting information on the archaeological information of the early colonial burial ground from Campeche’s central plaza is provided in the supplementary documentation of S1. In S2, the techniques of skeletal studies are presented. Previously conducted isotopic research procedures are documented in S3, while the results of radiocarbon dating of two of the ten samples are described in S4. The detailed methods of ancient DNA research used to generate the new paleogenomic information presented in this paper are outlined in S5. Dataset S1 provides extended information from ancient DNA analysis, Dataset S2 contains results of *qpAdm* analysis, and Dataset S3 provides the f_3_ statistics from comparison of Burial 11-1 to other African populations.

**S1. Context Information**

The following section is adapted from Tiesler et al. (2010) and reprinted here in edited form to provide further details of the procedures used to excavate samples selected for ancient DNA analysis: The individuals sampled for this study were recovered between January and June 2000 during rescue excavations of Campeche’s central plaza. Conceived as such, the excavation grid was laid out surrounding the foundations of the *Cabildo* Palace to be reconstructed. Two excavation units were established: one covered the area occupied by the Palace and a second one was assigned to the northeastern corner of the plaza. Exposure and recovery proceeded according to layers, trenches, and test pits. The first author coordinated the recording and recovery of the skeletal remains and was assisted by the personnel of the state INAH Center and students of the *Escuela Nacional de Antropología e Historia* (ENAH), and the *Facultad de Ciencias Antropológicas* (UADY). Subsequent restoration and investigation of the human remains were conducted first at the state INAH Center and then at the University of Yucatán in Mérida.

A total of five layers were excavated, revealing that the plaza gradually rose through time (Tiesler & Zabala 2010). The human interments under study were found in layers II, III and IV, distinguished by earth tone and compactness, degree of hydration and artifactual concentration. These fills accumulated centuries ago and harbored isolated and highly comingled cultural material datable to colonial times (mostly sherds), some of which was associated directly with the human remains. As the burial pits are intrusive, we presume that their stratigraphy provided merely a rough indication of the interments’ maximum depth more than any chronological range. Unfortunately, the burial plots, most of which show advanced decrees of commingling, were indistinguishable from the surrounding soil matrix and therefore did not provide any elements for stratigraphic sequencing. The sequence of layers II, III and IV is interrupted in a number of quadrants due to disturbances caused by more recent canalization excavation and park remodeling.

While the burial arrangements display a fairly uniform pattern when the layers are compared, their distribution and depth relative to each other permit a tentative sequencing. The shallowest burial intrusions (layer II), almost exclusively occupy the remote areas of the churchyard. The mortuary occupation of layer III also covers distant sections of the churchyard but concentrates in the intermediate spaces, which are also the most overcrowded in terms of burial density and disturbances. Opposite to the first two layers, layer IV occupation is solely found adjacent to the parish foundations and appears mostly undisturbed.

Two explanations could account for the spatial differences between the layers. The skeletons found in layer IV may have been interred in deeper pits than those excavated for the other deceased. However, a second explanation is much more likely, given the distinctive average distances of each layer relative to the church and the archival proof of the plaza’s repeated remodeling and fill (Antochiw 2010). Following this second assumption, the central square’s ground level rose gradually during the first two centuries of its existence. Thus, shallower burials are more recent in time while deeper burials are older.

No evidence of social segmentation (family units or other social groupings) was found amongst the burials surrounding the church (Brothwell 1987; S1c), suggesting a lack of social hierarchy. The only discernable pattern from previous analysis denotes that isotopically non-local and epigenetically distant individuals tended to be buried further from the church, suggesting that their interment was more recent than those closer to the church (Price et al. 2012; Scott and Turner 1997).

Interestingly, the material record from the cemetery is noted for the lack of any proper clothing accessories, like buttons, crosses, buckles, needles, belt hooks, clothing fasteners or any other popular ornament (Deagan 2002). Since most of these were made of durable material like metal alloys, stone or bone, we would expect their presence despite deterioration, if they had been there. Their absence indicates therefore that the mortal’s clothing was very humble or that the deceased were buried merely covered by a shroud, emulating the austere way Jesus Christ was buried, as was popular during these times.

Very few proper burial goods, apart from sets of beads potentially used for rosaries (Cucina *et al.* 2005) and one devotional medal, were found. As expected for a Christian cemetery, personal attire was rare and none would qualify as “offering” in the Prehispanic sense. Beads counted among the personal belongings that accompanied seven burials, although most of them could not be ascribed with certainty to any particular individual due to disturbances or their commingled nature. The shape and color identify a number of these items as black jet beads, which made their appearance in the Spanish colonies during the middle of the XVII^th^ century (Deagan 1987: 182-183). A further devotional medal from this same context was made of a copper alloy and with flanges projecting from the sides. Similarly flanged medals have been described for early colonial shipwreck and frontier assemblages dated before 1650 (Deagan 2002: 47-50).

The interpretation of primary and secondary interments (with their disturbances) was founded on an analysis of human archaeothanatology (Duday *et al.* 2009). All but 18 of the 129 burials excavated displayed signs of disturbance (Figure S1). The low social profile inferred from the associated material record is consistent with the lack tombstones, crosses, or other grave markers in this archaeological assemblage, none of which were to be found with the bodies in the area under exploration. Our results indicate that there was no orderly fashion burial deposition, which would have made memory of exact prior burial placement difficult. In practice, this lack of demarcation should have led to disturbances of older burials as new burials were placed in the cemetery, as it was expanding away from the church.

*Inventory of the ten burials under ancient DNA examination.* The petrous segments used for the paleogenetic study (see also S5) were sampled from both primary and secondary burials, prioritizing state of preservation and non-local provenience, as inferred from Sr, O, and C isotopic profiles (Figures S2 and S3). The distribution of these ten burials spans the occupational space of the burial ground. While Burial 128 was located within the confines of the church foundations (inside and probably near the altar), the remaining nine specimens were recovered from the surrounding atrium. Applying distance and depth to this cohort, it seems likely that Burial 30, Burial 52, and Burial 128, all documented in Level IV and located close to or inside the church, should have been placed relatively early during the cemetery in function, while the remainder, more distant and shallower graves, should correspond to more recent placements.

The skeletal drawings of the six sampled primary skeletons are represented in Figure S3. As illustrated, all skeletons (Burial 30, Burial 52-1, Burial 60, Burial 89, Burial 106-1, and Burial 128-1, the associated remains of which were analysed for this work) represent simple pit graves into which the bodies had been lowered and extended on their backs, the arms crossed over the thorax or abdomen. Each primary burial had been disturbed at some point afterwards, probably long after having skeletonized. Either the cranium or segments of the lower extremities were missing. All but one of the six burials in this category included associated isolated or partly articulated remains of additional individuals.

The schematic drawings of four more commingled and secondary assemblages are represented in the bottom row of Figure S3. As shown, the distribution of each one (Burial 18-2, Burial 78, and Burial 124 sec.) shows extensive disturbances or lack of articulation, indicating complete or almost complete removal from the original interment space after skeletonization. All four burials in this class displayed segments of additional individual(s), or themselves appear spatially associated to primary burials. It is of note that in Burial 78, diagnostic features of chronic venereal syphilis were recorded in postcranial segments, although these do not necessarily belong to the individual examined in this study. Among the individuals of the commingled multiple Burial 124 (one of which was analyzed in this study as Burial 124 sec.), counts an African foreigner according to isotopic signatures and African borne dental modification patterns. This individual had been buried with a necklace and a medallion.

**S2. Conventional Skeletal Analyses**

The following section is adapted from Price *et al.* (2012) and is reprinted here in edited form to provide details of the procedures used in morphological assessment of all individuals from the burial ground including those selected for ancient DNA analysis in this study. The skeletal analysis of these 129 burials retrieved from the colonial burial grounds in 2000 was conducted at the Laboratory of Bioarcheology of the Autonomous University of Yucatán, Mexico. Basic taphonomic assessment was founded on the principles of French-borne archaeothanatology (Duday 2009). It included estimation of minimum numbers of individuals, general state of preservation, and anatomic representation. Assessment of articulation patterns and effects of constriction were applied to the reconstruction of potential burial methods and shrouding. Individual anatomical representation of the overall series ranges from 1 to close to 100 percent but was in most cases well under the 10 percent threshold due to poor preservation and the post-interment disturbances described in S1.

*Sex estimation.* Sex was determined in all adults and late adolescents (from about 15 years of age and up), using available primary and secondary dimorphic features in the pelvis and the skull (Buikstra & Ubelaker 1994). Additional measurements on long bones diaphysis, epiphyses, and talus segments were combined by using regression formulae founded on sexed Maya reference populations (Tiesler 1999; Wrobel *et al.* 2002). In the absence of primary diagnostic features, all sex estimations were expressed as ‘probable’.

*Age-at-death estimates.* Subadult age at death determinations were founded on the successive stages of dental eruption, growth, and maturation in the deciduous and permanent dentition. In older subadult classes, stage of epiphyseal fusion was additionally taken into account, as described in Hartwig (1990); Buikstra & Ubelaker (1994). Adult age-at-death was estimated from primary diagnostic morphological features of the pelvis when present (auricular surface and pubic symphysis). Examination of additional diagnostic attributes were weighted in the absence of primary attributes, such as the degree of cranial suture closure and general degenerative patterns, as described in Buikstra & Ubelaker (1994). The degree of dental wear of the occlusal molar surfaces was systematically recorded in the overall series by adapting the taxonomy set forth by Brothwell (1987). When none of the diagnostic segments were present, burials were classified as ‘infant’, ‘child’, ‘adolescent’, or ‘adult’.

*Pathologies.* Well preserved bones from articulated individuals were subjected to macroscopic and histomorphologic pathological examination, founded on the parameters described in Buikstra and Ubelaker (1994), Ortner (2003), and Schultz (1988, 2003). Thin sections of selected diseased bone segments were processed at the facilities of the Laboratory of Bioarchaeology of the Autonomous University of Yucatán and followed the protocol published in Tiesler *et al.* (2006).

*Discrete dental traits.* Prior to this study, population affinity was ascribed exclusively on the study of discrete dental traits due to the poor condition of much of the skeletal material by Andrea Cucina (Cucina *et al.* 2005; Appendix 3 of Tiesler *et al.* 2010). Ancestries were assigned based on the frequencies of dental traits reported for African populations (Scott & Turner 1997) and native Maya (Cucina *et al.* 2005). Final ancestry assignments were made for 92 individuals using discrete dental traits and, when available, further criteria derived from the absence versus presence of artificial dental and cranial vault modifications (Tiesler & Oliva 2010), and isotopically inferred dietary patterns (Balasse *et al.* (2002); Koch *et al.* (1997). Some 25 individuals of the 92 ascribed individuals were classified as having potential African origin, 43 native, 19 ‘mestizo’, and 5 European (Tiesler *et al.* 2010: Appendix 3). The published assessments for the ten individuals in this paleogenetic study were determined to be either “probable [?]” or “possible [??]”.

*Permanent body modifications.* Some 43 cranial vaults were evaluated regarding their cultural cranial modification by using criteria established for classifying this practice among Mesoamerican skeletal populations (Romano 1965; Tiesler 2014); of those, only seven had discernable modifications, effected in all cases with cradle boards, a common practice among the Maya at the time of European conquest and beyond (Tiesler & Oliva 2010: 132). Dental modifications (Romero 1958; Tiesler 2002) were recorded among five out of 54 individuals analyzed. These five individuals displayed signs of artificial tooth reduction in the form of grooves on the incisal surface, systematic fracturing by way of chipping (precision blows) and dental filing. The latter two methods have been reported by a number of authors for African-borne populations (Handler *et al.* 1982; Milner & Larsen 1991; Tiesler & Olivia Arias 2010).

**S3. Isotopic Sample Preparation and Measurement**

Geographic origins and mobility, along with the dietary profiles of the individuals unearthed from the cemetery, were determined through isotopic analyses led by T. Douglas Price and his team at the University of Wisconsin-Madison and reported in Price *et al.* 2012 (see also Price 2014 for a description of methods). One hundred skeletal individuals were selected in this burial series on the grounds of dental and long-bone preservation. For a description of the methods and steps taken in isotopic analysis see Supplement C of Price *et al.* (2012). The isotopic study consisted of lead isotopes measured in 10 enamel samples, bone samples from 51 individuals, four bone samples from individuals without associated tooth samples. Bone samples are cortical bone from mid-length of femur in every case. Carbon isotope ratios were measured in 41 samples of bone were, 51 samples of bone apatite, and 64 samples of enamel apatite. Nitrogen isotope ratios were recorded in 41 samples of bone collagen. Oxygen isotopes were measured in 51 samples of bone apatite and 64 samples of enamel apatite. Each of these samples represents one individual.

In addition, strontium, carbon, and oxygen isotopes in tooth enamel were measured from nine individuals from two other colonial churches in Campeche for comparison. Strontium, carbon, and oxygen isotopes were measured in human tooth enamel, and strontium isotopes were measured in six animal bones from archaeological sites in Andalucía, Spain, for baseline information from one of the potential homelands of the inhabitants of Campeche.

*Strontium*. Campeche sits in an area of Eocene limestone with a geological strontium isotope ratio of approximately 0.7077. Local marine sediments in this coastal region might have higher isotope ratios approaching 0.7092, the value for modern seawater. Thus, from the geology alone we would predict strontium isotope ratios for Campeche to lie between 0.7077 and 0.7092. The site of Champoton, 65 km southwest of Campeche along the Gulf Coast, is the closest locality from which we have other baseline data and should be similar to Campeche; it has an average ^87^Sr/^86^Sr value of 0.7083.

Samples from two *barrio* churches from colonial Campeche were analyzed for comparison to the plaza cemetery. These individuals are thought to be locally born inhabitants of Campeche. Five human enamel samples from the Iglesia de San Roman on the south side of the town averaged 0.7083; four human enamel samples from the Rescate San Francisco on the north side of Campeche averaged 0.7085. Both sets of samples were highly homogeneous. These values likely provide a good estimate of the baseline strontium isotope ratio for local individuals born in or near Campeche. Samples of enamel and bone carbonate from the Campeche cemetery were analyzed using a multiple-collector thermal ionization mass spectrometer (TIMS).

*Lead.* There is still not much information on geographic variation in lead isotopes ratios across Mesoamerica. We also know, on the basis of samples analyzed to date, that there is very little lead in the sedimentary rocks of the lowland Maya area or in the teeth of the local inhabitants of Campeche. Lead was measured in the ultraclean lab facilities of Robert Frei at the University of Copenhagen using an established protocol.

*Light Isotopes: Carbon and Nitrogen*. The measurement of carbon isotope ratios in bone collagen is well known in the study of marine resources and C_4_ plants in human diets (e.g., Schoeninger & DeNiro 1984; Tauber 1981; van der Merwe & Vogel 1978). The method has been in use for a number of years and is well established and standard procedures were used in the study of Campeche individuals (Price et al. 2012).

*Oxygen.* Oxygen has three isotopes, ^16^O (99.762%), ^17^O (0.038%), and ^18^O (0.2%), all of which are stable and nonradiogenic. Oxygen isotopes are much lighter and have a much greater relative mass difference than strontium isotopes (^18^O is 12% heavier than ^16^O; ^87^Sr is 1% heavier than ^86^Sr), making them, in sharp contrast to strontium, highly sensitive to environmental and biological processes. Oxygen isotopes have been employed in a number of studies in Mesoamerica (e.g., Spence *et al.* 2005; White *et al.* 2001; White *et al.* 2002, 2004; White *et al.* 1998, 2000; Wright & Schwarcz 1998) and standard protocols were used to examine the Campeche individuals (Price et al. 2012).

*Carbon and Nitrogen in Bone Collagen.* Well-established procedures for extracting bone collagen, and bone and tooth enamel apatite were performed in the Laboratory for Archaeological Science at the University of South Florida. The average d^13^C collagen value (-9.3‰) is quite positive relative to the accepted range between pure C_3_ diets of approximately -21.5‰ and primary consumption of C_4_ plants such as maize and/or marine foods, with values of -14.0‰ or less negative. The average d^15^N value for the bone collagen samples (-9.3‰) is not significantly elevated, implying that seafood was at most a minor component of the diet, despite the location of Campeche, an important fishing harbor today, on the western coast of the Yucatán Peninsula (Price et al. 2012).

*Carbon Isotopes in Enamel Apatite.* d^18^O and d^13^C of tooth enamel carbonate were measured by David Dettman at the University of Arizona on the carbonate fraction of dental enamel using an automated carbonate preparation device (KIEL-III) coupled to a gas-ratio mass spectrometer (Finnigan MAT 252).

*Oxygen Isotopes in Apatite.* Histograms of d^18^O values in enamel and bone apatite from the Campeche burials were used to explore the variation present in these ratios and to see if we could distinguish individuals from different homelands. The shape of the distributions suggests that there is little significant variation present. Only one individual of the 29 for whom both measurements were made, likely an African, has a bone-enamel d^18^O shift greater than 2.5‰. This individual also has a significant bone-enamel ^87^Sr/^86^Sr shift from 0.7126 to the local ratio of 0.7087, suggesting arrival in the New World as a young individual. Oxygen isotopes in this study generally failed to provide diagnostic information for determining geographic origin (Price et al. 2012).

**S4. Radiocarbon Dating**

Radiocarbon dating is problematical in remains from the Colonial era, given that they are too recent to provide reliable and narrow chronological distribution plots. Two of the ten individuals were radiocarbon-dated on the grounds of representativeness of the time bracket of burial occupation: Burial 18-2 and Burial 124 (see also further radiocarbon dates, reported in Price *et al.* 2012: 399-402). The depth of both burials (1.34 m and 1.29 m) is slightly deeper that the collective average of burial depth of 1.15m (Tiesler & Zabala 2010) and therefore less prone to be disturbed. Both specimens were recovered from a middle layer right in the core area of mortuary occupation. From the above we infer parsimoniously that both dates should fall within the time bracket of occupation and not be outliers.

The samples in this project were dated at the Pennsylvania State University radiocarbon laboratory. The calibration of C14 dates to calendar years follows the published standards (Reimer *et al.* 2013; Ramsey 2017). The calibrated date ranges (2 sigma) encompass the decades just before Spanish contact, which we parsimoniously ignore, given the historical information and the idea that the archaeological burial disposition of both individuals followed post-contact, Catholic burial traditions. The more recent segment of both curves covers almost the complete span of time during which the cemetery was in use. The trends of the calibrated date ranges obtained for this study conform to previous assessments of ten individuals from the same cemetery, the values of which fall within the historically known period of occupation (Price *et al.* 2012).

**S5. Ancient DNA Analysis**

*Permissions and Ethics*

Permission for sampling, research, and exportation was obtained from the Consejo de Arqueología of the Instituto Nacional de Antropología e Historia (INAH) on March 2017 (Oficio No. 401.3S.16-2017/290; and 401-3-2201; exp. AA_08-17). This is the Mexican federal entity legally responsible for issuing research permits. In addition, we carried out public consultation with professional collaborators from Campeche and Yucatán. Results in this paper are part of a larger collaboration between the Reich Laboratory and Dr. Tiesler, funded in part by the National Geographic (2019-2021) and supported by a standing agreement between Harvard Medical School and the Autonomous University of Yucatán, undersigned in 2020. As part of this project we have conducted local engagement for the purpose of dissemination, namely at the historic center of the city of Campeche and the Yucatecan Maya communities of Dzemul and Yaxunah **(the latter being co-financed by the Yaxuná Archaeological Project (PIPCY) of the University of California at Riverside.**

*Methods*

Ancient DNA analyses were conducted on the petrous portion of the temporal bone for each individual in this study. The petrous portion has been demonstrated to provide up to one hundred times more ancient DNA per milligram than any other skeletal element (Pinhasi *et al.* 2015). This was reflected in the results showing that we were able to successfully acquire working ancient DNA from all the samples on which we attempted analysis. At least 40,000 single-nucleotide polymorphisms (SNPs) were covered by at least one sequence in every sample, and in all but one individual at least 500,000 SNPs were covered. Such success rates are impressive, particularly for warm environments in which DNA degrades rapidly.

Each sampled petrous portion was separated and packed individually at the Laboratory of Bioarchaeology, UADY, Mexico, after lateralization and pre-shipment documentation had been completed and the permission for destructive analyses obtained from the INAH Consejo de Arqueología of Mexico. Upon completion of ancient DNA sampling the petrous portions were returned to UADY. The laboratory used its standard protocol to sample the petrous portions. Processing of the petrous began in one of the clean rooms dedicated to sampling of ancient DNA. These rooms have a number of features, such as positive air pressure, UVC light sterilization, and wearing of coverall, two layers of gloves, facemask, and visor by technicians to minimize contamination from modern DNA.

In the clean room, petrous bones are sampled using a sandblaster. Samples are first “cleaned” with sand to remove the superficial exterior surface of the bone, which exposure to the depositional environment or previous handling could contaminate. Bone surrounding the cochlea is then removed (Pinhasi *et al.* 2019). The cochlea is then extracted, cleaned (again with the sandblaster), and then milled into a fine powder in a mixer mill (Retsch). This powder is then turned into an extract (approximately 75 mg of bone powder were used for each DNA extraction). This process involves the dissolution of bone power in a solution, which is followed by a series of previously described steps to isolate, clean and amplify the DNA (Dabney *et al.* 2013; Rohland *et al.* 2015). The samples show the typical damage profiles for ancient samples treated in this way, demonstrating that ancient DNA was successfully extracted (Figure S4).

Following Fu *et al.* (2013, 2015b), approximately 1.2 million SNPs were targeted, sequenced on a NextSeq500 Illumina instrument, and mapped to the human genome reference sequence *hg19* and to the mitochondrial genome reference sequence *rsrs* as previously described (Mathieson *et al.* 2015). For the whole genome data, an allele at each position was determined by randomly sampling a single position. For the mitochondrial DNA, a consensus sequence was built.

To examine the relatedness of the Campeche individuals to other Native American groups we created a neighbor-joining tree with “outgroup-*f_3_*” statistics of the form *f_3_(Mbuti; Pop1, Pop2)*, where Pop1 and Pop2 were either the Native American Campeche individuals, modern Mexican individuals, or ancient Indigenous California Channel Island individuals. The Campeche individuals grouped closest with modern Yucatán Maya individuals. To further examine the ancestry of Burial 11-1 we performed ordered statistics of the form f_3_(South_Africa_2000BP; Mexico_Colonial_African, African_modern). Dataset S3 provides the results of this test.

**Materials and Methods in Ancient DNA Research**

**DNA extraction, library preparation, in-solution enrichment and sequencing**: We followed previously published procedures for processing petrous samples (Pinhasi *et al.* 2019), extracting DNA (Dabney *et al.* 2013; Korlević *et al.* 2015), preparing libraries for partial UDG treatment, removing characteristic damage except at the final nucleotides (Rohland *et al.* 2015), and enriching for approximately 1.24 million single nucleotide polymorphism (SNP) targets and also for the mitochondrial genome (Fu *et al.* 2013, 2015a; Haak *et al.* 2015; Mathieson *et al.* 2015).

**Alignments and identification of DNA**: Raw sequences were trimmed of identification artifacts and aligned separately to both the human reference genome (*hg19*) and to the mitochondrial RSRS genome (Behar *et al.* 2012), removing duplicate molecules as needed. The mitochondrial genome alignments were used to determine haplogroups and contamination estimates; all other analyses used the human reference alignments. At the 1.24 million known polymorphic sites, low quality sequences (including those with indels) were discarded requiring minimum mapping quality and base quality values of 10 and 20 respectively, calling a random base from filtered sequences for each sample at each position where possible.

**Contamination Estimates:** mtDNA contamination was estimated using *schmutzi* (parameters: --notusepredC --uselength) (Renaud *et al.* 2015). For male individuals, X-chromosome contamination was estimated using ANGSD (Korneliussen *et al.* 2014) with the parameters: minimum base quality=20, minimum mapping quality=30, bases to clip for damage=2, and set all other parameters to the default. No samples were removed based on these analyses. All contamination estimates are reported in supplemental dataset S1.

**MtDNA haplogroups**: For mtDNA, the program *haplogrep2* was used to determine a haplogroup from a consensus sequence built for each sample (Weissensteiner *et al.* 2016).

**Genetic Sex***:* This was determined by computing Y = the number of sequences overlapping SNPs on the Y chromosome and X = the number of sequences overlapping SNPs on the X chromosomes. We then computed the ratio R=Y/(X+Y). Individuals with R<0.03 were labeled as female and individuals with R>0.35 were labeled as male based on study of this ratio in other samples for which similar data has been generated.

**Y chromosome haplogroup**: For Y chromosome haplogroup calling, we went back to the individual sequences (BAM-files) and performed an independent processing procedure. We filtered reads with mapping quality < 30 and bases with base quality < 30 and trimmed the first and last 2bp of each sequence to remove potential errors due to characteristic ancient DNA damage. We determined the most derived mutation for each individual using the tree of the International Society of Genetic Genealogy (ISOGG) version 11.110 (accessed 21 April 2016) and confirmed the presence of upstream mutations consistent with the assigned Y chromosome haplogroup using *Yfitter* (Jostins *et al.* 2014).

**ADMIXTURE:** We first used *PLINK2* (Chang *et al.* 2015) to prune the dataset using the –geno 0.7 option to remove all sites that had less than 70% of the samples with a called genotype. We then ran *ADMIXTURE* (Alexander *et al.* 2009) with K=2 to 6 with 10 replicates for each K. We ran ADMIXTURE in unsupervised mode, which, as an unsupervised clustering method, can make mistakes for smaller sample sizes and slightly closer ancestry (French is more similar to Native American relative to West African), explaining the apparent Native American ancestry in French individuals in Figure 5. We thus view ADMIXTURE as more of a qualitative method that shows a general result that we then followed up with more rigorous analyses (qpAdm).

**Principal Component Analysis:** Principal component analysis was performed using *smartPCA* in EIGENSOFT (Patterson *et al.* 2012). We used the default parameters except for “lsqproject: YES” and performed PCA on the modern East Asian (Han Chinese), African (Yoruba and Mende), European (French and Finnish), and Native American (Pima, Chane, and Mixe) individuals from the SGDP dataset (Mallick *et al.* 2016). We then projected ancient individuals on the principal components determined using present-day individuals.

***qpAdm* Analysis:** We performed formal modeling of the Campeche individuals by using *qpAdm* software (Haak *et al.* 2015). We first considered each Campeche individual as a target along with Mixe, Yoruba, and French as ‘source’ populations; together these were assigned as “left” populations to determine the ancestry proportions for each of the Campeche. We used the following right set (outgroup populations) of previously published data to distinguish the different components of the Campeche individuals: USA-MT_Anzick1_12800BP, Ethiopia_4500BP (Gallego Llorente *et al.* 2015), and Kostenki14 (Seguin-Orlando *et al.* 2014). We used the parameter “details: YES”, which reports a normally distributed p-value for the fit (estimated with a block jackknife). In all cases the Campeche individual could be fit well as mixtures in various combinations of the three sources of ancestry (p>0.01). We also modeled each individual with pairs of potential surrogates for the source populations (Mixe-Yoruba, Mixe-French, Yoruba-French), and with single source surrogates (Mixe, Yoruba, or French), adding the individual not modeled each time to the set of right groups. This demonstrated that the Native American Campeche individuals could fit as clades with Mixe, that the African Campeche individual could fit as a clade with Yoruba, and that the European Campeche individual could fit as a clade with French (as a stand-in for European).

**Symmetry Statistic (*f*-statistics) Based Analyses:** We used the *qp3pop* and *qpDstat* packages in ADMIXTOOLS (Patterson *et al.* 2012) to compute *f_3_*-statistics and *f_4_*-statistics (using the f4Mode: YES parameter in *qpDstat*) and computed standard errors with a weighted block jackknife over 5-Mb blocks. We used the inbreed: YES parameter when we computed *f_3_*-statistics to account for our random allele calling at each position. We computed “outgroup” *f_3_*-statistics of the form *f_3_(Mbuti; Pop1, Pop2)*, which measures the shared genetic drift between Pop1 and Pop2. We created a matrix of the outgroup-*f_3_* values between all pairs of populations. We converted the original values to distances by taking the inverse of the values and generated a neighbor joining tree using PHYLIP version 3.696’s (Felsenstein 1989) neighbor function and setting *USA-CA_SanNicolas_4000BP* as the outgroup (default settings were used for the rest of the analysis). We displayed the tree using *Itol* and set all of the tree lengths to ignore (Letunic & Bork 2011).

**Additional Analysis for Burial 11-1**

We computed statistics of the form *D(Mbuti, Test; Yoruba, Mexico_Colonial_African)* for Burial 11-1. These were not significantly positive suggesting an absence of Native or European ancestry (if Native American or European ancestry were present, then when *Test* was either Native American or European, it would be significantly positive). We also computed outgroup-*f_3_* statistics of the form *f_3_(SouthAfrica_2000BP; Mexico_Colonial_African, African_SGDP_modern),* where *SouthAfrica_2000BP* was an ancient South African known to be an outgroup to modern Africans (Skoglund *et al*. 2017) and *African_SGDP_modern* were different modern African groups throughout West, Central, South, and East Africa. The groups that maximized the statistic had the highest shared drift with *Mexico_Colonial_African* and were *Yoruba, Esan, Igbo,* and *Lemande* (in that order; Online Dataset S3).

**Data Availability**

All raw data are available at the European Nucleotide Archive and the National Center for Biotechnology Information under the accession number PRJEB50901 and at https://reich.hms.harvard.edu/datasets.

**Supplement Figures and Figure Legend**

**
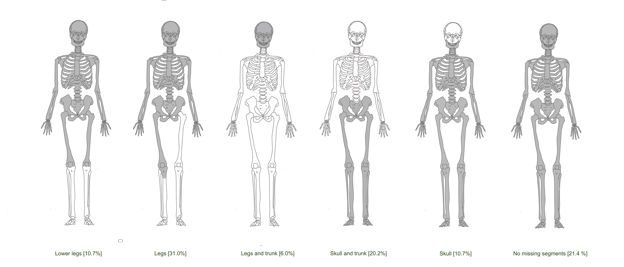
**

**Figure S1.** Proportions of articulated skeletons with missing anatomical segments due to the frequent reoccupation of the burial ground, as recorded during recovery in 2000 (Centro INAH Campeche, INAH).


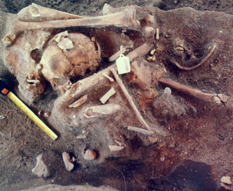

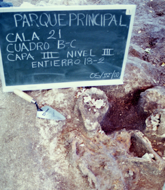

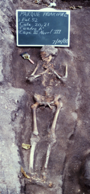

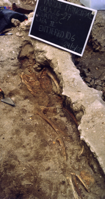


**Figure S2.** Burials 11-1, 18-2, 52-1, and 106-1 during on-site recording and recovery in 2000 (Centro INAH Campeche, INAH).

**
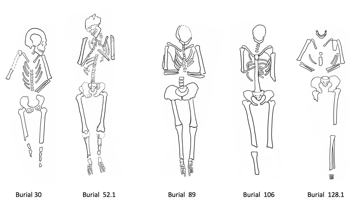

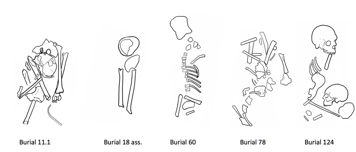
**

**Figure S3.** Individual skeletal tracings of the ten burials under study. The top row displays five primary undisturbed or slightly reduced deposits. The bottom row represents the five heavily disturbed primary burials and completely disarticulated, commingled (secondary) human remains included in this study.

**
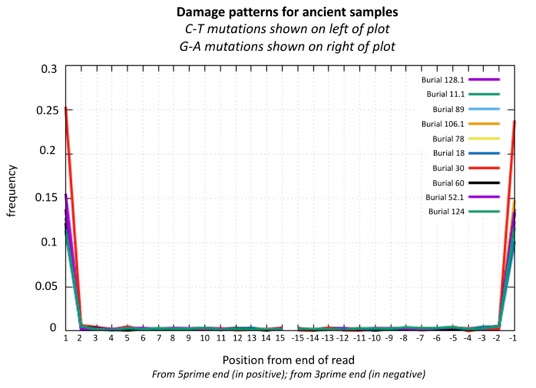
**

**Figure S4.** Deamination profiles for newly sequenced samples.

**References**

ALEXANDER, D.H., J. NOVEMBRE & K. LANGE. 2009. Fast model-based

estimation of ancestry in unrelated individuals. Genome Research 19: 1655–64. https://doi.org/10.1101/gr.094052.109.

ALT, K.W. 1997. Odontologische Verwandtschaftsanalyse: Individuelle

Charakteristika der Zähne in ihrer Bedeutung für Anthropologie, Archäologie und Rechtsmedizin. Stuttgart: Gustav Fischer.

BALASSE, M., et al. 2002. The Seasonal Mobility Model for Prehistoric Herders

in the South-western Cape of South Africa Assessed by Isotopic Analysis of Sheep Tooth Enamel. Journal of Archaeological Science 29: 917–32. https://doi.org/10.1006/jasc.2001.0787.

BEHAR, D.M., et al. 2012. A “Copernican” Reassessment of the Human

Mitochondrial DNA Tree from its Root. The American Journal of Human

Genetics 90: 675–84. https://doi.org/10.1016/j.ajhg.2012.03.002.

BROTHWELL, D.R. 1987. Desenterrando huesos. Mexico City: Fondo de

Cultura Económica.

BUIKSTRA, J.E. & D. UBELAKER (ed.). 1994. Standards for Data Collection

from Human Skeletal Remains: Proceedings of a Seminar at the Field

Museum of Natural History (Arkansas Archeological Report Research Series) by Buikstra, Jane E. (1994) Paperback. Vol. Arkansas Archaeological Survey Research Series no. 44. Fayetteville, AR: Arkansas Archeological Survey.

CHANG, C.C., et al. 2015. Second-generation PLINK: rising to the challenge of

larger and richer datasets. GigaScience 4: 7.

https://doi.org/10.1186/s13742-015-0047-8.

CUCINA, A., V. TIESLER & G. WROBEL. 2005. Afinidades biológicas y

dinámicas poblacionales Mayas desde el Clásico hasta el periodo colonial. Los Investigadores de la Cultural Maya 13: 559–67.

DABNEY, J. et al. 2013. Complete mitochondrial genome sequence of a Middle

Pleistocene cave bear reconstructed from ultrashort DNA fragments. Proceedings of the National Academy of Sciences 110: 15758–63. https://doi.org/10.1073/pnas.1314445110.

DUDAY, H., A.M. CIPRIANI & J. PEARCE. 2009. The Archaeology of the

Dead: Lectures in Archaeothanatology. Oxford : Oakville, CT: Oxbow Books.

ELNESR, N.M., AND J.K. AVERY. 1994. Tooth eruption and shedding, in J. K.

Avery (ed.) Oral development and histology: 110–129. New York: Thieme Medical Publishers.

FELSENSTEIN, J. 1989. PHYLIP - Phylogeny Inference Package (Version 3.2).

Cladistics 5: 164–66.

FU, Q., et al. 2013. DNA analysis of an early modern human from Tianyuan

Cave, China. Proceedings of the National Academy of Sciences 110:

2223–27. https://doi.org/10.1073/pnas.1221359110.

FU, Q. et al. 2015a. An early modern human from Romania with a recent

Neanderthal ancestor. Nature 524: 216–19.

https://doi.org/10.1038/nature14558.

—. 2015b. An early modern human from Romania with a recent Neanderthal ancestor. Nature 524: 216–19. https://doi.org/10.1038/nature14558.

GALLEGO LLORENTE, M. et al. 2015. Ancient Ethiopian genome reveals

extensive Eurasian admixture throughout the African continent. Science (New York, N.Y.) 350: 820–22. https://doi.org/10.1126/science.aad2879.

HAAK, W. et al. 2015. Massive migration from the steppe was a source for Indo-

European languages in Europe. Nature 522: 207–11.

https://doi.org/10.1038/nature14317.

HANDLER, J.S., R.S. CORRUCCINI & R.J. MUTAW. 1982. Tooth mutilation

in the Caribbean: Evidence from a slave burial population in barbados.

Journal of Human Evolution 11: 297–313. https://doi.org/10.1016/S00472484(82)80021-3.

HARTWIG, W.C. 1990. The anatomy and biology of the human skeleton by D.

Gentry Steele and Claud A. Bramblett. Texas A & M University Press,

College Station, 1988. Clinical Anatomy 3: 151–53.

https://doi.org/10.1002/ca.980030210.

HILLSON, S. 2005. Teeth. Cambridge: Cambridge University Press.

JOSTINS, L., et al. 2014. YFitter: Maximum likelihood assignment of Y

chromosome haplogroups from low-coverage sequence data. arXiv:1407.7988 [q-bio]. http://arxiv.org/abs/1407.7988.

KOCH, P.L., N. TUROSS & M.L. FOGEL. 1997. The Effects of Sample

Treatment and Diagenesis on the Isotopic Integrity of Carbonate in

Biogenic Hydroxylapatite. Journal of Archaeological Science 24: 417–29. https://doi.org/10.1006/jasc.1996.0126.

KORLEVIĆ, P., et al. 2015. Reducing microbial and human contamination in

DNA extractions from ancient bones and teeth. BioTechniques 59: 87–93. https://doi.org/10.2144/000114320.

KORNELIUSSEN, T.S., A. ALBRECHTSEN & R. NIELSEN. 2014. ANGSD:

Analysis of Next Generation Sequencing Data. BMC Bioinformatics 15:

356. https://doi.org/10.1186/s12859-014-0356-4.

LETUNIC, I. & P. BORK. 2011. Interactive Tree Of Life v2: online annotation

and display of phylogenetic trees made easy. Nucleic Acids Research 39:

W475-478. https://doi.org/10.1093/nar/gkr201.

MALLICK, S. et al. 2016. The Simons Genome Diversity Project: 300 genomes

from 142 diverse populations. Nature 538: 201–6.

https://doi.org/10.1038/nature18964.

MATHIESON, I. et al. 2015. Genome-wide patterns of selection in 230 ancient

Eurasians. Nature 528: 499–503. https://doi.org/10.1038/nature16152.

MILNER, G.R. & C.S. LARSEN. 1991. Teeth As Artifacts of Human Behavior:

Intentional Mutilation and Accidental Modification, in M.A. Kelley & C.S. Larsen (ed.) Advances In Dental Anthropology: 357–78. Wiley-Liss.

PATTERSON, N., et al. 2012. Ancient Admixture in Human History. Genetics

192: 1065–93. https://doi.org/10.1534/genetics.112.145037.

PINHASI, R. et al. 2015. Optimal Ancient DNA Yields from the Inner Ear Part of

the Human Petrous Bone. (ed.) M.D. Petraglia PLOS ONE 10: e0129102.

https://doi.org/10.1371/journal.pone.0129102.

PINHASI, R., et el. 2019. Isolating the human cochlea to generate bone powder

for ancient DNA analysis. Nature Protocols.

<https://doi.org/10.1038/s41596-019-0137-7>.

PRICE, T.D. 2014. An Introduction to the Isotopic Studies of Ancient Human

Remains. Journal of the North Atlantic, Special Volume 7: 71–87.

PRICE, T.D., et al. 2012. Isotopic Studies of Human Skeletal Remains from a

Sixteenth to Seventeenth Century AD Churchyard in Campeche, Mexico:

Diet, Place of Origin, and Age. Current Anthropology 53: 396–433. https://doi.org/10.1086/666492.

RAMSEY, C.B. 2017. Methods for Summarizing Radiocarbon Datasets.

Radiocarbon 59: 1809–33. https://doi.org/10.1017/RDC.2017.108.

REIMER, P.J. et al. 2013. IntCal13 and Marine13 Radiocarbon Age Calibration

Curves 0–50,000 Years cal BP. Radiocarbon 55: 1869–87.

RENAUD, G., et al. 2015. Schmutzi: estimation of contamination and

endogenous mitochondrial consensus calling for ancient DNA. Genome

Biology 16. <http://genomebiology.com/2015/16/1/224>.

https://doi.org/10.1186/s13059-015-0776-0.

ROHLAND, N., et al. 2015. Partial uracil-DNA-glycosylase treatment for

screening of ancient DNA. Philosophical Transactions of the Royal

Society of London. Series B, Biological Sciences 370: 20130624. https://doi.org/10.1098/rstb.2013.0624.

ROMANO, A. 1965. Estudio morfológico de la deformación craneana en Tamuín,

S.L.P., y en la Isla del Idolo, Veracruz. Mexico City: Instituto Nacional de

Antropología e Historia.

ROMERO, J. 1958. Mutilaciones dentarias prehispánicas de México y América

en general. Mexico City: Instituto Nacional de Antropología e Historia.

SCHOENINGER, M.J., AND M.J. DENIRO. 1984. Nitrogen and carbon iso-

topic composition of bone collagen from marine and terrestrial animals. Geochimica et Cosmochimica Acta 48: 625–639.

SCOTT, G.R. & C.G. TURNER. 1997. The Anthropology of Modern Teeth.

Dental Morphology and its Variation in Recent Human Poulations.

(Cambridge Studies in Biological Anthropology). Cambridge: Cambridge

University Press.

SEGUIN-ORLANDO, A. et al. 2014. Genomic structure in Europeans dating

back at least 36,200 years. Science 346: 1113–18.

https://doi.org/10.1126/science.aaa0114.

SKOGLUND, P. et al. 2017. Reconstructing Prehistoric African Population

Structure. Cell 171: 59-71.e21. https://doi.org/10.1016/j.cell.2017.08.049.

SPENCE, M.W., et al. 2005. Past lives in different places: the origins and

relationships of Teotihuacan’s foreign residents, in R. E. Blanton (ed.)

Settlement, subsistence, and social complexity: essays honoring the legacy

of Jeffrey R. Parsons: 155–197. Los Angeles: Cotsen Institute of

Archaeology.

TIESLER, V. 1999. Rasgos bioculturales entre los antiguos mayas: aspectos

arqueológicos y sociales. PhD, Mexico City: Universidad Nacional

Autónoma de México.

TIESLER, V. 2002. New cases of an African tooth decoration from colonial

Campeche, Mexico. Homo: Internationale Zeitschrift Fur Die

Vergleichende Forschung Am Menschen 52: 277–82.

TIESLER, V. 2014. The Bioarchaeology of Artificial Cranial Modifications. New

Approaches to Head Shaping and its Meanings in Pre-Columbian

Mesoamerica and Beyond. New York: Springer Press.

TIESLER, V. & I. OLIVIA ARIAS. 2010. Identity, Alienation, and Integration:

Body Modifications in the Early Colonial Population from Campeche, in

V. Tiesler, P. Zabala & A. Cucina (ed.) Natives, Europeans, and Africans

in Colonial Campeche: History and Archaeology: 130–51. Gainesville,

FL: University Press of Florida.

TIESLER, V., P. ZABALA & A. CUCINA. 2010. Natives, Europeans, and

Africans in Colonial Campeche: History and Archaeology. University

Press of Florida. https://muse.jhu.edu/book/17597.

VAN DER MERWE, N.J., AND J.C. VOGEL. 1978. 13C content of human

collagen as a measure of prehistoric diet in woodland North America.

Nature 276: 815–816.

WEISSENSTEINER, H., et al. 2016. HaploGrep 2: mitochondrial haplogroup

classification in the era of high-throughput sequencing. Nucleic Acids

Research 44: W58-63. https://doi.org/10.1093/nar/gkw233.

WHITE, C.D., et al. 1998. Oxygen isotopes and the identification of geo-

graphical origins: the Valley of Oaxaca versus the Valley of Mexico. Journal of ¬Archaeological Science 25: 643–655.

WHITE, C., F.J. LONGSTAFFE, AND K.R. LAW. 2001. Revisiting the

Teotihuacan connection at Altun Ha: oxygen-isotope analysis of Tomb f-

8/1. Ancient Mesoamerica 12: 65–72.

WHITE, C.D., M.W. SPENCE, AND F.J. LONGSTAFFE. 2002. Geographic

identities of the sacrificial victims at the Feathered Serpent Pyramid: implications for the nature of state power. Latin American Antiquity 13: 217–236.

WHITE, C.D., M.W. SPENCE, AND F.J. LONGSTAFFE. 2004. Demography

and ethnic continuity in the Tlailotlacan enclave of Teotihuacan: the

evidence from stable oxygen isotopes. Journal of Anthropological Archaeology 23: 385–403.

WRIGHT, L.E., AND H.P. SCHWARCZ. 1998. Stable carbon and oxygen

isotopes in human tooth enamel: identifying breastfeeding and weaning in

prehistory. American Journal of Physical Anthropology 106: 1–18.

WROBEL, G.D., M.E. DANFORTH & C. ARMSTRONG. 2002. Estimating sex

of Maya skeletons by discriminant function analysis of long-bone measurements from the protohistoric maya site of Tipu, Belize. Ancient Mesoamerica 13: 255–63. https://doi.org/10.1017/S0956536102132044.
