# Supplementary material for: Life and death in early colonial Campeche: new insights from ancient DNA
Source: Antiquity. Author manuscript; Available in PMC 2024 Dec 4. (PMC11617036; doi:10.15184/aqy.2022.79)
Supplement: Supplementary Table 1 [file NIHMS2033175-supplement-Supplementary_Table_1.pdf]

ONLINE TABLE 1 (SAMPLE INFO)

Reich Lab ID	Skeletal code	Location	Latitude	Longitude	Country	Analysis Label after quality control and formal clustering	Genetic ancestry vs. [macroscopic det. of ancestry]	Isotopically determined provenience	Cultural head shaping in infancy	Cultural dental modifications	Pathologies	Genetic sex compared to [macroscopic sex]	Mean date BP	Mean date BCE (or CE shown as negative numbers)	Date range (either 95% confidence interval from a direct date or from archaeological context	14C date	Skeletal element	Location where skeletal sample was prepared into powder	Location where extraction and library preparation were performed	Skeletal sampling technique
I8555	#1 Burial 18-2	Campeche, Campeche	20.24	-90.46	México	Mexico_Colonial	AME [NID]	[NID]	[NID]	[NID]	-	F [M?]	390	-1560	1481-1639 calC	Yes	petrous	Boston	Boston	sandblast
I8556	#2 Burial 30	Campeche, Campeche	20.24	-90.46	México	Mexico_Colonial	AME [AME?]	Local	[NID]	None	-	F [NID]	340	-1610	1540-1680 CE	No direct date	petrous	Boston	Boston	sandblast
I8557	#3 Burial 60	Campeche, Campeche	20.24	-90.46	México	Mexico_Colonial	AME [AFR??]	Local	None	None	-	F [NID]	340	-1610	1540-1680 CE	No direct date	petrous	Boston	Boston	sandblast
I8558	#4 Burial 52	Campeche, Campeche	20.24	-90.46	México	Mexico_Colonial_o	EUR [EUR?/AFR?]	Non local	None	Facets from pipe use	Periostosis, enamel hypoplasia	M [M?]	340	-1610	1540-1680 CE	No direct date	petrous	Boston	Boston	sandblast
I8559	#5 Burial 124-1	Campeche, Campeche	20.24	-90.46	México	Mexico_Colonial	AME [AME?]	[NID]	Yes	[NID]	-	M [NID]	415	-1535	1446-1624 calC	Yes	petrous	Boston	Boston	sandblast
I10859	#6 Burial 128	Campeche, Campeche	20.24	-90.46	México	Mexico_Colonial	AME [NID]	Local	None	[NID]	-	F [M?]	340	-1610	1540-1680 CE	No direct date	petrous	Boston	Boston	sandblast
I10860	#7 Burial 11-1	Campeche, Campeche	20.24	-90.46	México	Mexico_Colonial_African	AFR [AFR; AME?]	Non local	None	[NID]	Enamel hypoplasia	F [F?]	340	-1610	1540-1680 CE	No direct date	petrous	Boston	Boston	sandblast
I10861	#8 Burial 89	Campeche, Campeche	20.24	-90.46	México	Mexico_Colonial	AME [NID]	Local	None	[NID]	-	M [M?]	340	-1610	1540-1680 CE	No direct date	petrous	Boston	Boston	sandblast
I10862	#9 Burial 106	Campeche, Campeche	20.24	-90.46	México	Mexico_Colonial	AME [NID]	Local	None	[NID]	Periostosis, porotic hyperostosis, enamel hypoplasia	M [NID]	340	-1610	1540-1680 CE	No direct date	petrous	Boston	Boston	sandblast
I10863	#10 Burial 78	Campeche, Campeche	20.24	-90.46	México	Mexico_Colonial	AME [NID]	Local	None	[NID]	Possibly veneral syphilis	F [F?]	340	-1610	1540-1680 CE	No direct date	petrous	Boston	Boston	sandblast

Reich Lab ID	mg powder used in extraction	DNA extraction references	Library ID(s)	UDG Treatment	mg sample powder per library	Library preparation references	Target enrichment experiments performed	Percentage of sequences in library mapping to human reference genome	mtDNA coverage (by library)	mtDNA damage rate in first nucleotide (for sequences mapping to the mtDNA genome, by library)	95% confidence interval for mtDNA match to the consensus sequence (by library)	mtDNA haplogroup (restricting to calls with haplogrep2 rank scoes of at least 0.7)	1240K enrichment damage rate in first nucleotide for sequences mapping to the human genome (per library)	1240K Coverage (cumulative if > 1 library)	1240K SNPs hit on autosomes (cumulative if > 1 library)	Sex determination (from 1240K data)	Y-chromosome haplogroup (from 1240K data)	X-chromosome contamination estimate (from 1240K data)	X-chromosome contamination Z-score from zero (from 1240K data)	Detected as a relatives of others in the dataset within the third degree? (from 1240K data)	Nature of newly published data
I8555	77	Dabney 2013, Korlevic 2015	S8555.E1.L1	half	8.6	Robotic UDGhalf	1240k, mtDNA+3000SNPs	0.0876	28	0.112	[0.976,0.996]	A2+(64)	0.104	1.998402	766523	F		0.207813	40.96456649	n/a	entirely new
I8556	68	Dabney 2013, Korlevic 2015	S8556.E1.L1	half	7.6	Robotic UDGhalf	1240k, mtDNA+3000SNPs	0.0155	9.98	0.251	[0.975,0.999]	A2r	0.13	0.035657	40807	F		0.132031	0.995308824	n/a	entirely new
I8557	76	Dabney 2013, Korlevic 2015	S8557.E1.L1	half	8.4	Robotic UDGhalf	1240k, mtDNA+3000SNPs	0.049	34	0.133	[0.982,0.998]	A2+(64)	0.115	1.266702	661696	F		0.178664	29.83550262	n/a	entirely new
I8558	65	Dabney 2013, Korlevic 2015	S8558.E1.L1	half	7.2	Robotic UDGhalf	1240k, mtDNA+3000SNPs	0.1725	48.1	0.142	[0.990,1.000]	X2d2	0.121	2.833017	845074	M	I2a2a1b	0.005402	4.254303722	n/a	entirely new
I8559	77	Dabney 2013, Korlevic 2015	S8559.E1.L1	half	8.6	Robotic UDGhalf	1240k, mtDNA+3000SNPs	0.0783	45	0.121	[0.979,0.995]	R	0.109	1.94572	798004	M	Q1a2a1a1	0.004454	3.092160118	n/a	entirely new
I10859	77	Dabney 2013, Korlevic 2015	S10859.E1.L1	half	8.6	Robotic UDGhalf	1240k+	0.14352535	783.9	0.162	[0.984,0.997]	B2	0.172	2.347498	730884	F				n/a	entirely new
I10860	72	Dabney 2013, Korlevic 2015	S10860.E1.L1	half	8.0	Robotic UDGhalf	1240k+	0.050037463	285.6	0.133	[0.980,0.996]	L3e2b+152	0.144	0.983449	587322	F				n/a	entirely new
I10861	75	Dabney 2013, Korlevic 2015	S10861.E1.L1	half	8.3	Robotic UDGhalf	1240k+	0.050466442	270.4	0.124	[0.973,0.991]	B2+16278	0.135	1.241291	669233	M	Q1a2a1a1	0.00894	4.063636364	n/a	entirely new
I10862	74	Dabney 2013, Korlevic 2015	S10862.E1.L1	half	8.2	Robotic UDGhalf	1240k+	0.033076987	227.7	0.145	[0.959,0.980]	A2	0.152	0.904746	565042	M	Q1a2a1a	0.0033	1.571428571	n/a	entirely new
I10863	71	Dabney 2013, Korlevic 2015	S10863.E1.L1	half	7.9	Robotic UDGhalf	1240k+	0.473416726	695.9	0.129	[0.984,0.996]	A2m	0.138	3.255336	776783	F				n/a	entirely new

References

Method	Abbreviation	References and technical modifications
Extraction	Dabney 2013	J. Dabney *et al.* , Complete mitochondrial genome sequence of a Middle Pleistocene cave bear reconstructed from ultrashort DNA fragments. *Proc. Natl. Acad. Sci. U. S. A.* **110**, 15758–63 (2013).
Extraction	Korlevic 2015	P. Korlevic *et al.* , Reducing microbial and human contamination in DNA extractions from ancient bones and teeth. *Biotechniques* . **59**, 87–93 (2015).
Library preparation	Robotic UDGhalf	N. Rohland, E. Harney, S. Mallick, S. Nordenfelt, D. Reich, Partial uracil-DNA-glycosylase treatment for screening of ancient DNA. *Philos. Trans. R. Soc. B Biol. Sci.* 370, 20130624 (2015); silica caoted magnetic beads and 7x volume PB (Qiagen) for cleanup
Enrichment experiments performed	1240k, mtDNA+3C	separate enrichment for mtDNA and ~3000 nuclear targets (Olalde 2018 or Lipson 2018), and ~1.2 million SNPs (Fu et al., 2015; Haak et al., 2015; Mathieson et al., 2015)
Enrichment experiments performed	1240k+	simultaneous enrichment of ~1.2M SNPs and mtDNA (Fu et al., 2015; Haak et al., 2015; Mathieson et al., 2015, with 3bp tiled mitochondrial genome rsrs spike in)

ONLINE TABLE 2 (14C DATES)

PSUAMS#	Burial #	Reich ID	Site	Country	Material	Process	fraction Modern	±	D^{14}C (‰)	±	^{14}C age (BP)	±	_^{13}C (‰)	_^{15}N (‰)	%C	%N	C:N	Cal BCE/CE (_)	2_ low	2_ high
4430	#1 Burial 18-2	I8555	Campeche	Mexico	Petrous	XAD amino acids	0.9590	0.0020	-41.0	2.0	335	20	-7.1	9.5	24.4	8.8	3.22	1615	1481	1639
4431	#5 Burial 124-1	I8559	Campeche	Mexico	Petrous	>30kDa gelatin	0.9539	0.0019	-46.1	1.9	380	20	-7.7	8.5	13.3	4.7	3.27	1570	1446	1624

ONLINE TABLE 3 (LIST OF SAMPLES USED IN ANALYSIS)

Ancient Individuals

Population Label	Individual ID	Reference
USA_MT_Anzick1_12800BP.SG	Anzick	M. Rasmussen, *et al* ., Nature 2014
Peru_Lauricocha_8600BP	I0041	C. Posth, *et al* ., Cell 2018
	I0238	C. Posth, *et al* ., Cell 2018
	I0038	C. Posth, *et al* ., Cell 2018
Ethiopia_4500BP.SG	I5950	M. Gallego Llorente, *et al* ., Science 2014
Kostenki14	Kostenki14	A. Seguin-Orlando, *et al* ., Science 2014

Modern Individuals

Population Label	Individual ID	Reference
Yoruba.DG	HGDP00932	S. Mallick, *et al* ., Nature 2016
Yoruba.DG	HGDP00936	S. Mallick, *et al* ., Nature 2016
Yoruba.DG	HGDP00928	S. Mallick, et al., Nature 2016
Esan.DG	HG03100	S. Mallick, et al., Nature 2016
Esan.DG	HG02943	S. Mallick, et al., Nature 2016
Igbo	S_Igbo-2.DG	S. Mallick, et al., Nature 2016
Igbo	S_Igbo-1.DG	S. Mallick, et al., Nature 2016
Lemande.DG	S_Lemande-1	S. Mallick, et al., Nature 2016
Lemande.DG	S_Lemande-2	S. Mallick, et al., Nature 2016
Saharawi.DG	S_Saharawi-1	S. Mallick, et al., Nature 2016
Saharawi.DG	S_Saharawi-2	S. Mallick, et al., Nature 2016
Gambian.DG	HG02464	S. Mallick, et al., Nature 2016
Gambian.DG	HG02574	S. Mallick, et al., Nature 2016
Luhya.DG	NA19044	S. Mallick, et al., Nature 2016
Luhya.DG	NA19023	S. Mallick, et al., Nature 2016
Kongo.DG	S_Kongo-2	S. Mallick, et al., Nature 2016
BantuKenya.DG	HGDP01417	S. Mallick, et al., Nature 2016
BantuKenya.DG	HGDP01414	S. Mallick, et al., Nature 2016
Luo.DG	Ayodo_502C	S. Mallick, et al., Nature 2016
Luo.DG	Ayodo_430C	S. Mallick, et al., Nature 2016
Dinka.DG	A_Dinka-4	S. Mallick, et al., Nature 2016
Dinka.DG	DNK11	S. Mallick, et al., Nature 2016
Dinka.DG	DNK05	S. Mallick, et al., Nature 2016
Dinka.DG	DNK07	S. Mallick, et al., Nature 2016
Mende.DG	HG03078	S. Mallick, et al., Nature 2016
Mende.DG	HG03085	S. Mallick, et al., Nature 2016
Somali.DG	Ayodo_81S	S. Mallick, et al., Nature 2016
Masai.DG	NA21490	S. Mallick, et al., Nature 2016
Masai.DG	NA21581	S. Mallick, et al., Nature 2016
BantuHerero.DG	HGDP01035	S. Mallick, et al., Nature 2016
BantuHerero.DG	HGDP01028	S. Mallick, et al., Nature 2016
Biaka.DG	HGDP00461	S. Mallick, et al., Nature 2016
Biaka.DG	HGDP00457	S. Mallick, et al., Nature 2016
BantuTswana.DG	HGDP01030	S. Mallick, et al., Nature 2016
BantuTswana.DG	HGDP01034	S. Mallick, et al., Nature 2016
Mbuti.DG	HGDP00449	S. Mallick, et al., Nature 2016
Mbuti.DG	HGDP00476	S. Mallick, et al., Nature 2016
Mbuti.DG	HGDP00474	S. Mallick, et al., Nature 2016
Ju_hoan_North.DG	HGDP00987	S. Mallick, et al., Nature 2016
Ju_hoan_North.DG	HGDP00991	S. Mallick, et al., Nature 2016
Ju_hoan_North.DG	HGDP01032	S. Mallick, et al., Nature 2016
Khomani_San.DG	S_Khomani_S	S. Mallick, et al., Nature 2016
Khomani_San.DG	S_Khomani_S	S. Mallick, et al., Nature 2016
French.DG	HGDP00530	S. Mallick, et al., Nature 2016
French.DG	HGDP00526	S. Mallick, et al., Nature 2016
Mixe.DG	S_Mixe-2	S. Mallick, et al., Nature 2016
Mixe.DG	S_Mixe-3	S. Mallick, et al., Nature 2016
