# Supplementary material for: Life and death in early colonial Campeche: new insights from ancient DNA
Source: Antiquity. Author manuscript; Available in PMC 2024 Dec 4. (PMC11617036; doi:10.15184/aqy.2022.79)
Supplement: Supplementary Table 2 [file NIHMS2033175-supplement-Supplementary_Table_2.pdf]

ONLINE TABLE 2 (14C DATES)

PSUAMS#	Burial #	Reich ID	Site	Country	Material	Process	fraction Modern	±	$D^{14}C$ (‰)	±	^{14}C age (BP)	±	_^{13}C (‰)	_^{15}N (‰)	%C	%N	C:N	Cal BCE/CE (_)	2_ low	2_ high
4430	#1 Burial 18-2	I8555	Campeche	Mexico	Petrous	XAD amino acids	0.9590	0.0020	-41.0	2.0	335	20	-7.1	9.5	24.4	8.8	3.22	1615	1481	1639
4431	#5 Burial 124-1	I8559	Campeche	Mexico	Petrous	>30kDa gelatin	0.9539	0.0019	-46.1	1.9	380	20	-7.7	8.5	13.3	4.7	3.27	1570	1446	1624
