# Supplementary material for: Life and death in early colonial Campeche: new insights from ancient DNA
Source: Antiquity. Author manuscript; Available in PMC 2024 Dec 4. (PMC11617036; doi:10.15184/aqy.2022.79)
Supplement: Supplementary Table 3 [file NIHMS2033175-supplement-Supplementary_Table_3.pdf]

ONLINE TABLE 3 (LIST OF SAMPLES USED IN ANALYSIS)

Ancient Individuals

**Population Label**	**Individual ID**	**Reference**
USA_MT_Anzick1_12800BP.SG	Anzick	M. Rasmussen, *et al* ., Nature 2014
Peru_Lauricocha_8600BP	I0041	C. Posth, *et al* ., Cell 2018
	I0238	C. Posth, *et al* ., Cell 2018
	I0038	C. Posth, *et al* ., Cell 2018
Ethiopia_4500BP.SG	I5950	M. Gallego Llorente, *et al* ., Science 2014
Kostenki14	Kostenki14	A. Seguin-Orlando, *et al* ., Science 2014

Modern Individuals

**Population Label**	**Individual ID**	**Reference**
Yoruba.DG	HGDP00932	S. Mallick, *et al* ., Nature 2016
Yoruba.DG	HGDP00936	S. Mallick, *et al* ., Nature 2016
Yoruba.DG	HGDP00928	S. Mallick, et al., Nature 2016
Esan.DG	HG03100	S. Mallick, et al., Nature 2016
Esan.DG	HG02943	S. Mallick, et al., Nature 2016
Igbo	S_Igbo-2.DG	S. Mallick, et al., Nature 2016
Igbo	S_Igbo-1.DG	S. Mallick, et al., Nature 2016
Lemande.DG	S_Lemande-1	S. Mallick, et al., Nature 2016
Lemande.DG	S_Lemande-2	S. Mallick, et al., Nature 2016
Saharawi.DG	S_Saharawi-1	S. Mallick, et al., Nature 2016
Saharawi.DG	S_Saharawi-2	S. Mallick, et al., Nature 2016
Gambian.DG	HG02464	S. Mallick, et al., Nature 2016
Gambian.DG	HG02574	S. Mallick, et al., Nature 2016
Luhya.DG	NA19044	S. Mallick, et al., Nature 2016
Luhya.DG	NA19023	S. Mallick, et al., Nature 2016
Kongo.DG	S_Kongo-2	S. Mallick, et al., Nature 2016
BantuKenya.DG	HGDP01417	S. Mallick, et al., Nature 2016
BantuKenya.DG	HGDP01414	S. Mallick, et al., Nature 2016
Luo.DG	Ayodo_502C	S. Mallick, et al., Nature 2016
Luo.DG	Ayodo_430C	S. Mallick, et al., Nature 2016
Dinka.DG	A_Dinka-4	S. Mallick, et al., Nature 2016
Dinka.DG	DNK11	S. Mallick, et al., Nature 2016
Dinka.DG	DNK05	S. Mallick, et al., Nature 2016
Dinka.DG	DNK07	S. Mallick, et al., Nature 2016
Mende.DG	HG03078	S. Mallick, et al., Nature 2016
Mende.DG	HG03085	S. Mallick, et al., Nature 2016
Somali.DG	Ayodo_81S	S. Mallick, et al., Nature 2016
Masai.DG	NA21490	S. Mallick, et al., Nature 2016
Masai.DG	NA21581	S. Mallick, et al., Nature 2016
BantuHerero.DG	HGDP01035	S. Mallick, et al., Nature 2016
BantuHerero.DG	HGDP01028	S. Mallick, et al., Nature 2016
Biaka.DG	HGDP00461	S. Mallick, et al., Nature 2016
Biaka.DG	HGDP00457	S. Mallick, et al., Nature 2016
BantuTswana.DG	HGDP01030	S. Mallick, et al., Nature 2016
BantuTswana.DG	HGDP01034	S. Mallick, et al., Nature 2016
Mbuti.DG	HGDP00449	S. Mallick, et al., Nature 2016
Mbuti.DG	HGDP00476	S. Mallick, et al., Nature 2016
Mbuti.DG	HGDP00474	S. Mallick, et al., Nature 2016
Ju_hoan_North.DG	HGDP00987	S. Mallick, et al., Nature 2016
Ju_hoan_North.DG	HGDP00991	S. Mallick, et al., Nature 2016
Ju_hoan_North.DG	HGDP01032	S. Mallick, et al., Nature 2016
Khomani_San.DG	S_Khomani_S	S. Mallick, et al., Nature 2016
Khomani_San.DG	S_Khomani_S	S. Mallick, et al., Nature 2016
French.DG	HGDP00530	S. Mallick, et al., Nature 2016
French.DG	HGDP00526	S. Mallick, et al., Nature 2016
Mixe.DG	S_Mixe-2	S. Mallick, et al., Nature 2016
Mixe.DG	S_Mixe-3	S. Mallick, et al., Nature 2016
